# Comparison of cardiovascular outcomes between SGLT2 inhibitors in diabetes mellitus

**DOI:** 10.1186/s12933-022-01508-6

**Published:** 2022-05-18

**Authors:** Yuta Suzuki, Hidehiro Kaneko, Akira Okada, Hidetaka Itoh, Satoshi Matsuoka, Katsuhito Fujiu, Nobuaki Michihata, Taisuke Jo, Norifumi Takeda, Hiroyuki Morita, Kentaro Kamiya, Atsuhiko Matsunaga, Junya Ako, Koichi Node, Hideo Yasunaga, Issei Komuro

**Affiliations:** 1 The Department of Cardiovascular Medicine, The University of Tokyo, 7-3-1, Hongo, Bunkyo-ku, Tokyo, 113-8655 Japan; 2 Department of Rehabilitation Sciences, Kitasato University Graduate School of Medical Sciences, Sagamihara, Japan; 3 The Department of Advanced Cardiology, The University of Tokyo, Tokyo, Japan; 4 Department of Prevention of Diabetes and Lifestyle-Related Diseases, Graduate School of Medicine, The University of Tokyo, Tokyo, Japan; 5 The Department of Health Services Research, The University of Tokyo, Tokyo, Japan; 6 Department of Cardiovascular Medicine, Kitasato University School of Medicine, Sagamihara, Japan; 7 Department of Cardiovascular Medicine, Saga University, Saga, Japan; 8 The Department of Clinical Epidemiology and Health Economics, School of Public Health, The University of Tokyo, Tokyo, Japan

**Keywords:** SGLT2 inhibitor, Cardiovascular disease, Diabetes mellitus

## Abstract

**Background:**

There have been scarce data comparing cardiovascular outcomes between individual sodium-glucose cotransporter-2 (SGLT2) inhibitors. We aimed to compare the subsequent cardiovascular risk between individual SGLT2 inhibitors.

**Methods:**

We analyzed 25,315 patients with diabetes mellitus (DM) newly taking SGLT2 inhibitors (empagliflozin: 5302, dapagliflozin: 4681, canagliflozin: 4411, other SGLT2 inhibitors: 10,921). We compared the risks of developing heart failure (HF), myocardial infarction (MI), angina pectoris (AP), stroke, and atrial fibrillation (AF) between individual SGLT2 inhibitors.

**Results:**

Median age was 52 years, and 82.5% were men. The median fasting plasma glucose and HbA1c levels were 149 (Q1-Q3:127–182) mg/dL and 7.5 (Q1-Q3:6.9–8.6) %. During a mean follow-up of 814 ± 591 days, 855 HF, 143 MI, 815 AP, 340 stroke, and 139 AF events were recorded. Compared with empagliflozin, the risk of developing HF, MI, AP, stroke, and AF was not significantly different in dapagliflozin, canagliflozin, and other SGLT inhibitors. For developing HF, compared with empagliflozin, hazard ratios of dapagliflozin, canagliflozin, and other SGLT2 inhibitors were 1.02 (95% confidence interval [CI] 0.81–1.27), 1.08 (95% CI 0.87–1.35), and 0.88 (95% CI 0.73–1.07), respectively. Wald tests showed that there was no significant difference in the risk of developing HF, MI, AP, stroke, and AF among individual SGLT2 inhibitors. We confirmed the robustness of these results through a multitude of sensitivity analyses.

**Conclusion:**

The risks for subsequent development of HF, MI, AP, stroke, and AF were comparable between individual SGLT2 inhibitors. This is the first study comparing the wide-range cardiovascular outcomes of patients with DM treated with individual SGLT2 inhibitors using large-scale real-world data.

**Supplementary Information:**

The online version contains supplementary material available at 10.1186/s12933-022-01508-6.

## Introduction

Sodium-glucose cotransporter-2 (SGLT2) inhibitors are oral antidiabetic drugs that promote the urinary excretion of glucose by inhibiting renal proximal tubules from reabsorbing glucose, thereby lowering plasma glucose levels. Randomized clinical trials have demonstrated that SGLT2 inhibitors can improve cardiovascular outcomes in patients with diabetes mellitus (DM) [1–3]. In particular, the effect of SGLT2 inhibitors on the prevention of heart failure (HF) events has attracted unprecedented interest. For example, empagliflozin is reported to reduce the risk of hospitalization due to HF by 35% in patients with type 2 DM compared to placebo group [1]. Through the accumulation of clinical evidence, SGLT2 inhibitor is currently considered to be a key drug for DM from the perspective of the prevention for future cardiovascular disease (CVD) event [4, 5]. Accordingly, the prescription rate of SGLT2 inhibitors for patients with DM is markedly increasing [6, 7]. For example, the percentage of patients with type 2 DM prescribed SGLT2 inhibitors increased from 3.8% in 2015 to 11.9% in 2019 in the United States [6]. However, the magnitude of cardiovascular benefit of individual SGLT2 inhibitors was not consistent between trials [1–3, 8]. Further, several studies reported the potential difference in pharmacological effects and outcomes between individual SGLT2 inhibitors mainly due to a variety of SGLT2 selectivity [9–13]. Nevertheless, there have been scarce data comparing the cardiovascular outcomes between individual SGLT2 inhibitors using real-world clinical data, and therefore, it remains unclear whether a class effect of SGLT2 inhibitors on cardiovascular outcomes is present. Given the widespread use of SGLT2 inhibitors in clinical practice, it is necessary to assess the comparability of cardiovascular outcomes among individual SGLT2 inhibitors. SGLT2 inhibitors were first launched in Japan in 2014, and there are currently six commercially available SGLT2 inhibitors (empagliflozin, dapagliflozin, canagliflozin, ipragliflozin, tofogliflozin, and luseogliflozin). Here, we analyzed a large-scale real-world dataset including approximately 25,000 patients with DM who were newly prescribed SGLT2 inhibitors. This study aimed to compare the subsequent cardiovascular risk, including HF, myocardial infarction (MI), angina pectoris (AP), stroke, and atrial fibrillation (AF), between individual SGLT2 inhibitors.

## Methods

### Study population

**Fig. 1:**
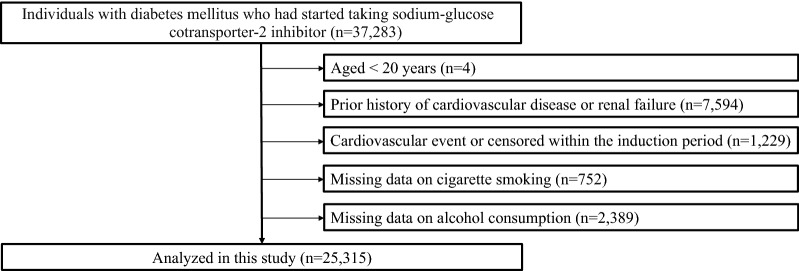
Flowchart. We extracted data on 37,283 individuals with diabetes mellitus (ICD-10 codes: E10−E14) who had started taking sodium-glucose cotransporter-2 inhibitors at least 4 months after enrollment (insurance coverage). Among them, we excluded individuals aged < 20 years (n = 4), those with a prior history of cardiovascular disease or renal failure (n = 7594), those who recorded any cardiovascular disease events or were censored within an induction period (1 month) (n = 1229), those with missing data on cigarette smoking (n = 752), and those with missing data on alcohol consumption (n = 2389). Finally, we analyzed 25,315 individuals in this study

We performed a retrospective cohort study using the JMDC Claims Database (JMDC Inc., Tokyo, Japan), which is a health check-up and insurance claims database, between January 2005 and April 2021 [14, 15]. The JMDC Claims Database includes individuals’ health checkup records, including data on BP, body mass index (BMI), medical history, current medications, and insurance claims data, including the diagnosis of CVD events according to the International Classification of Diseases, 10th Revision (ICD-10) coding. We extracted data from 37,283 individuals with DM (ICD-10 codes: E10−E14) who had started taking SGLT2 inhibitors at least 4 months after enrollment (insurance coverage). We set a 4 month look-back period because the maximum prescription period for medications is 3 months in Japan. We excluded individuals aged < 20 years (n = 4), those with a prior history of CVD or renal failure (n = 7594), those who recorded any CVD events or were censored within an induction period (1 month) (n = 1229), those with missing data on cigarette smoking (n = 752), and those with missing data on alcohol consumption (n = 2389). Finally, 25,315 individuals were analyzed in this study (Additional file 1: Figure S1 and Fig. 1).

### Ethics

This study was approved by the Ethics Committee of the University of Tokyo (Number: 2018-10862). This study was conducted in accordance with the Declaration of Helsinki. The requirement for informed consent was waived because all data included in this dataset were de-identified. Any person who purchases it from JMDC Inc. (https://www.jmdc.co.jp/en/index) can use this database.

### Measurements and definitions

We obtained health check-up data collected within 6 months before SGLT2 inhibitors were newly prescribed. We collected the following data: BMI, BP, fasting blood glucose, low-density lipoprotein cholesterol, high-density lipoprotein cholesterol, and triglycerides. If available, we also obtained data on HbA1c levels. We obtained information on cigarette smoking (current or non-current) and alcohol consumption (every day or not every day) from a self-reported questionnaire during the health check-up. From administrative claims records, we retrieved the presence of CVD (HF, MI, AP, stroke, and AF), renal failure (dialysis and kidney transplantation), and diabetic complications (nephropathy, retinopathy, and neuropathy) at the prescription date of SGLT2 inhibitors. Information on medications on the prescription date of SGLT2 inhibitors was also collected. Overweight/obesity was defined as BMI ≥ 25 kg/m^2^. Dyslipidemia was defined as low-density lipoprotein cholesterol ≥ 140 mg/dL, high-density lipoprotein cholesterol < 40 mg/dL, triglyceride ≥ 150 mg/dL, or use of lipid-lowering medications [16].

### Outcomes

The outcome data were obtained from January 2005 to April 2021. The primary outcomes included HF (ICD-10 codes: I500, I501, I509, and I110), MI (ICD-10 codes: I210–I214, and I219), AP (ICD-10 codes: I200, I201, I208, and I209), stroke (ICD-10 codes: I630, I631–I636, I638, I639, I600–I611, I613–I616, I619, I629, and G459), and AF (ICD-10 code: I480–I484, and I489) (separately).

### Statistical analysis

Continuous variables were presented as medians (interquartile range, quartile 1, and quartile 3), and categorical variables were described as numbers (percentages). We categorized the study participants by individual SGLT2 inhibitors (empagliflozin, dapagliflozin, canagliflozin, and other SGLT2 inhibitors). Considering prescription rates, sample sizes, and global approval status, we combined ipragliflozin, tofogliflozin, and luseogliflozin in one group. We calculated the statistical significance of the differences between groups using analysis of variance for continuous variables and chi-square tests for categorical variables. We conducted a Cox regression analysis to identify the association between individual SGLT2 inhibitors and the subsequent incidence of each CVD event. Empagliflozin was used as the reference. Model 1 included only individual SGLT2 inhibitors (unadjusted model). Model 2 included individual SGLT2 inhibitors, age, and sex, and multivariable Cox regression analyses (forced entry model) were conducted. Furthermore, as in Model 3, we added BMI, hypertension, fasting plasma glucose, dyslipidemia, cigarette smoking, alcohol consumption, diabetic nephropathy, diabetic retinopathy, diabetic neuropathy, insulin use, dipeptidyl peptidase-4 (DPP-4) inhibitor use,  glucagon-like peptide-1 (GLP-1) receptor agonist use, biguanide use, sulfonylurea use, α-glucosidase inhibitor use, thiazolidine use, glinide use, and year of prescription to Model 2, and we performed multivariable Cox regression analyses (forced entry model). We conducted the Wald test to compare the hazard ratios (HRs) between individual SGLT2 inhibitors.

Nine sensitivity analyses were performed to confirm the robustness of our findings. First, we examined the association between SGLT2 inhibitors and cardiovascular events only in individuals who continued to use the same SGLT2 inhibitor for over 3 months. Second, we prolonged the induction period from 30 to 90 days. Third, we conducted multiple imputations for missing data as previously described [15]. Fourth, since death should be considered a competing risk with CVD events, we performed cause-specific Cox proportional hazard modeling as a competing risks analysis [15]. Fifth, we compared the all-cause mortality among individual SGLT2 inhibitors. Sixth, the baseline estimated glomerular filtration rate (eGFR) was adjusted. Seventh, we analyzed individuals diagnosed with type 2 DM (ICD-10 code: E11). Eighth, we separately compared the cardiovascular outcomes between the six SGLT2 inhibitors. Finally, we conducted subgroup analyses stratified by age, sex, and baseline HbA1c levels. The null hypothesis was rejected for (two-tailed) values of p < 0.05. All statistical analyses were performed using Stata v17 (StataCorp LLC, College Station, TX, USA).

## Results

### Clinical characteristics

Table 1 summarizes the clinical characteristics of the study participants. Overall, the median age was 52 (47–58) years, and 20,875 (82.5%) patients were men. The median fasting plasma glucose and HbA1c levels were 149 (Q1-Q3 127–182) mg/dL and 7.5 (Q1–Q3 6.9–8.6) %, respectively. The prevalence of overweight/obesity, hypertension, and dyslipidemia were 75.9%, 58.9%, and 80.8%, respectively. Diabetic nephropathy, retinopathy, and neuropathy were observed in 15.4%, 22.8%, and 3.2% of the patients, respectively. DPP-4 inhibitors and insulin were used in more than half and 8.2% of the study participants, respectively. The study participants were categorized into four groups according to individual SGLT2 inhibitors: empagliflozin (n = 5302), dapagliflozin (n = 4681), canagliflozin (n = 4411), and other SGLT2 inhibitors (n = 10,921 and 5275 for ipragliflozin, 3074 for tofogliflozin, and 2572 for luseogliflozin).

**Table 1:** Baseline characteristics

	Overall (n = 25,315)	Empagliflozin (n = 5302)	Dapagliflozin (n = 4681)	Canagliflozin (n = 4411)	Other SGLT2-inihitibors (n = 10,921)	P-value
Age, years	52 (47–58)	52 (46–58)	52 (46–57)	52 (46–58)	52 (47–58)	0.037
Men, n (%)	20,875 (82.5)	4392 (82.8)	3838 (82.0)	3710 (84.1)	8935 (81.8)	0.005
Body mass index, kg/m^2^	27.8 (25.1–31.1)	27.8 (25.1–31.2)	27.9 (25.3–31.2)	27.7 (25.0–31.1)	27.7 (25.0–31.0)	0.025
SBP, mmHg	129 (120–140)	129 (120–140)	129 (120–140)	130 (121–139)	129 (120–140)	0.74
DBP, mmHg	82 (75–89)	82 (74–89)	82 (75–89)	82 (75–89)	82 (74–89)	0.52
Cigarette smoking, n (%)	8634 (34.1)	1761 (33.2)	1625 (34.7)	1526 (34.6)	3722 (34.1)	0.37
Alcohol consumption, n (%)	5106 (20.2)	1058 (20.0)	937 (20.0)	930 (21.1)	2181 (20.0)	0.43
Comorbidity						
Overweight/obesity, n (%)	19,205 (75.9)	4050 (76.4)	3626 (77.5)	3321 (75.3)	8208 (75.2)	0.011
Hypertension, n (%)	14,909 (58.9)	3099 (58.4)	2781 (59.4)	2619 (59.4)	6410 (58.7)	0.67
Dyslipidemia, n (%)	20,465 (80.8)	4314 (81.4)	3775 (80.6)	3570 (80.9)	8806 (80.6)	0.71
Diabetic nephropathy, n (%)	3889 (15.4)	892 (16.8)	629 (13.4)	639 (14.5)	1729 (15.8)	< 0.001
Diabetic retinopathy, n (%)	5770 (22.8)	1276 (24.1)	990 (21.1)	871 (19.7)	2633 (24.1)	< 0.001
Diabetic neuropathy, n (%)	804 (3.2)	181 (3.4)	142 (3.0)	124 (2.8)	357 (3.3)	0.32
Medication						
Insulins, n (%)	2081 (8.2)	444 (8.4)	446 (9.5)	278 (6.3)	913 (8.4)	< 0.001
DPP-4 inhibitor, n (%)	14,001 (55.3)	2876 (54.2)	2370 (50.6)	2510 (56.9)	6245 (57.2)	< 0.001
GLP-1 receptor agonist, n (%)	528 (2.1)	139 (2.6)	107 (2.3)	73 (1.7)	209 (1.9)	0.003
Biguanide, n (%)	11,723 (46.3)	2649 (50.0)	2089 (44.6)	1928 (43.7)	5057 (46.3)	< 0.001
Sulfonylurea, n (%)	4765 (18.8)	888 (16.7)	840 (17.9)	763 (17.3)	2274 (20.8)	< 0.001
α-glucosidase inhibitor, n (%)	2378 (9.4)	459 (8.7)	423 (9.0)	352 (8.0)	1144 (10.5)	< 0.001
Thiazolidine, n (%)	2392 (9.4)	406 (7.7)	450 (9.6)	376 (8.5)	1160 (10.6)	< 0.001
Glinides, n (%)	757 (3.0)	180 (3.4)	107 (2.3)	126 (2.9)	344 (3.1)	0.007
Laboratory data						
Glucose, mg/dL	149 (127–182)	149 (128–183)	148 (126–183)	147 (125–181)	149 (127–182)	0.011
HbA1c, %	7.5 (6.9–8.6)	7.5 (6.9–8.6)	7.5 (6.9–8.6)	7.5 (6.8–8.5)	7.6 (6.9–8.6)	0.001
LDL-C, mg/dL	123 (103–145)	123 (103–146)	124 (103–146)	124 (103–146)	122 (103–144)	0.076
HDL-C, mg/dL	49 (43–58)	49 (42–57)	49 (43–57)	49 (43–57)	49 (43–58)	0.079
Triglycerides, mg/dL	140 (99–206)	141 (100–210)	140 (99–206)	142 (102–206)	139 (97–203)	0.014

Data are reported as medians (interquartile range) or numbers (percentage), where appropriate

*SBP* systolic blood pressure, *DBP* diastolic blood pressure, *DPP-4* dipeptidyl peptidase-4, *GLP-1* glucagon-like peptide-1, *LDL-C* low-density lipoprotein cholesterol, *HDL-C* high-density lipoprotein cholesterol

### Risk of CVD events between SGLTS2 inhibitors

**Fig. 2:**
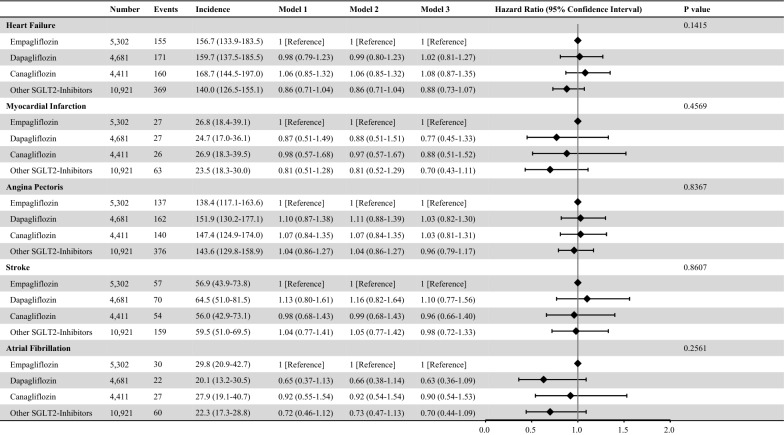
Risk of Cardiovascular Event among SGLT2 Inhibitors. We compared the risks of HF, MI, AP, stroke, and AF between individual SGLT2 inhibitors. Incidence rates were presented as per 10,000 person-years

During a mean follow-up period of 814 ± 591 days, 855 HF, 143 MI, 815 AP, 340 stroke, and 139 AF events were recorded. In the unadjusted model (Model 1), compared with empagliflozin, the risks of dapagliflozin, canagliflozin, and other SGLT2 inhibitors for HF, MI, AP, stroke, and AF were not statistically different. In the multivariable adjustment model (Model 3), compared to empagliflozin, the risk of developing HF, MI, AP, stroke, and AF did not differ between dapagliflozin, canagliflozin, and other SGLT2 inhibitors (Fig. 2). Wald tests showed that there was no significant difference in the risk of developing HF, MI, AP, stroke, or AF among individual SGLT2 inhibitors.

### Sensitivity analysis

**Fig. 3:**
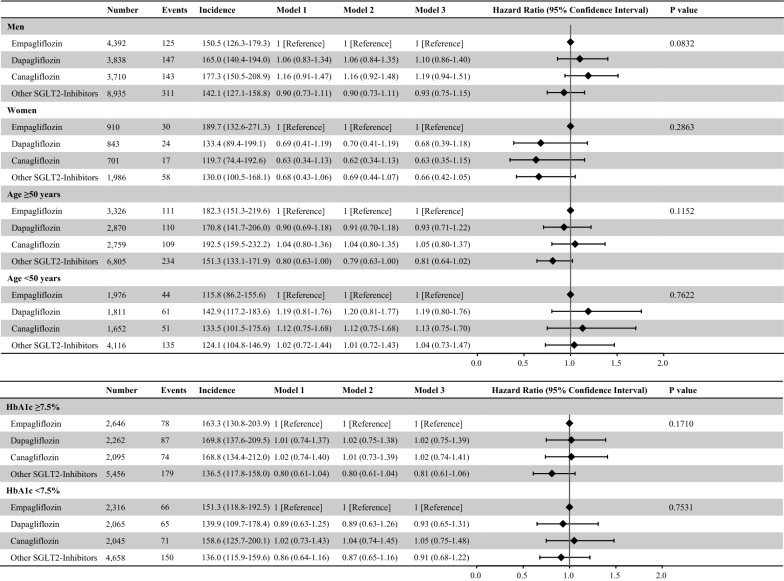
Risk of Heart Failure among SGLT2 Inhibitors (Subgroup analysis). We compared the risk of HF between individual SGLT2 inhibitors stratified by sex, age, and baseline HbA1c level. Sex was excluded from the adjusted variables in the subgroup analysis stratified by sex. We excluded 1772 individuals with missing HbA1c data from the subgroup analysis stratified by HbA1c level. Incidence rates were presented as per 10,000 person-years

First, the discontinuation rates 3 months after the first prescription were 18.7% for empagliflozin, 19.2% for dapagliflozin, 16.1% for canagliflozin, and 17.3% for other SGLT2 inhibitors. We included 20,819 participants who continued to use the same SGLT2 inhibitor over 3 months and found that the risk of developing each CVD event was comparable between individual SGLT2 inhibitors (Additional file 1: Figure S2). Second, we prolonged the induction period from 30 to 90 days and analyzed 23,688 patients. In this population, the risk of developing CVD was comparable between individual SGLT2 inhibitors (Additional file 1: Figure S3). Third, we analyzed 28,456 patients after multiple imputations for missing data, and there was no significant difference in the risk of CVD events among individual SGLT2 inhibitors in this population (Additional file 1: Figure S4). Fourth, our results remained unchanged after competing risks analysis (Additional file 1: Figure S5). Fifth, mortality risk did not differ among individual SGLT2 inhibitors (Additional file 1: Figure S6). Sixth, we analyzed 12,957 patients with available eGFR data. Even after adjustment for the eGFR, the risk of each CVD event was comparable between the individual SGLT2 inhibitors (Additional file 1: Figure S7). Seventh, we analyzed 18,194 participants with a diagnosis of type 2 DM and found that the risk for each CVD event did not differ between individual SGLT2 inhibitors (Additional file 1: Figure S8). Eighth, the risk of developing HF, MI, AP, stroke, and AF did not differ between the six individual SGLT2 inhibitors (Additional file 1: Figure S9). The risk of developing HF was comparable between individual SGLT2 inhibitors, irrespective of age, sex, or baseline HbA1c level (Fig. 3).

## Discussion

In the present study, we analyzed 25,315 patients with DM who had started taking SGLT2 inhibitors and demonstrated that there was no significant difference in the risk of developing HF, MI, AP, stroke, and AF among patients taking empagliflozin, dapagliflozin, canagliflozin, and other SGLT2 inhibitors. A variety of sensitivity analyses confirmed our results. To the best of our knowledge, this is the first study to compare the wide-range cardiovascular outcomes of patients with DM treated with individual SGLT2 inhibitors using large-scale real-world data.

DM increases the risk of various cardiovascular events (e.g., HF, coronary artery disease, AF) and cardiovascular deaths [17–19], and therefore, clinical trials demonstrating the robust cardiovascular benefits of SGLT2 inhibitors for patients with DM, revolutionized clinical practice [1–3]. The most noteworthy finding included the salutary effects of SGLT2 inhibitors on HF. SGLT2 inhibitors have also been reported to reduce atherosclerotic cardiovascular mortality and morbidity in patients with type 2 DM [1, 2, 20]. An analysis of new users of SGLT2 inhibitors showed that the use of SGLT2 inhibitors was associated with a lower risk of MI (HR: 0.81, 95% CI 0.74–0.88) and stroke (HR 0.68, 95% CI 0.55–0.84) [20]. SGLT2 inhibitors may prevent the development of AF [21, 22]. An analysis of DECLARE-TIMI 58 showed that dapagliflozin use reduced future risk of AF/atrial flutter by 19% in patients with type 2 DM [21]. While many studies on SGLT2 inhibitors and cardiovascular outcomes have been accumulated, there have been few data on comparisons of cardiovascular event risk among SGLT2 inhibitors. Recently, an analysis of a retrospective cohort in Taiwan compared the cardiovascular outcomes of dapagliflozin and empagliflozin, and showed that dapagliflozin might have a more favorable effect on HF than empagliflozin [9]. Another study reported that canagliflozin and empagliflozin were most effective for the prevention of HF hospitalization [10]. Therefore, whether the risk of cardiovascular events differs among individual SGLT2 inhibitors is attracting attention.

Our study is distinguishable from previous studies in that we compared the risk of developing specific cardiovascular outcomes in patients with DM between commercially available SGLT2 inhibitors using a large-scale real-world dataset in Japan and found that there was no significant difference in the risk of HF, MI, AP, stroke, and AF among individual SGLT2 inhibitors. Simultaneously, our study raises additional research questions. First, we analyzed patients with DM without a history of CVD in this study, and therefore, this study focuses on the cardiovascular outcomes in a primary prevention setting. Although the cardiovascular benefit of SGLT2 inhibitor use in primary prevention settings has been shown in various studies [20, 23, 24], it would be more evident in secondary prevention settings [8, 25]. Therefore, further investigations, including patients with DM and a history of CVD, are warranted to compare the cardiovascular outcomes between SGLT2 inhibitors in secondary CVD prevention settings. Second, SGLT2 inhibitors reduce not only cardiovascular events but also renal events [26–30]. However, clinical data comparing renal outcomes between SGLT2 inhibitors are scarce. Whether the benefit for renal outcomes would be different for each SGLT2 inhibitor is of clinical interest. Third, the potential adverse effects of SGLT2 inhibitors should not be ignored, and there are unresolved concerns regarding the specific adverse effects of SGLT2 inhibitor use (e.g., lower limb amputation, bone fracture, genitourinary infection, ketoacidosis) [2, 31, 32]. Although the discontinuation rates were similar between SGLT2 inhibitors, the incidence of these adverse clinical events needs to be compared between SGLT2 inhibitors. To confirm our findings and address the aforementioned issues, conducting a randomized clinical trial is an ideal solution. However, considering the required sample size, follow-up period, and costs, this would not be feasible in practice. Therefore, further analyses using epidemiological data (including administrative claims data, such as ours) are needed.

Our study has several strengths. We conducted this study using a large-scale administrative claims dataset, which provided a sufficient sample size for the statistical analysis. This large sample size allows a multitude of sensitivity analyses, which strengthens the robustness of our main result. Another advantage is the high retention of study participants in the JMDC claims database. The JMDC Claims Database includes insurance claims data; thus, all clinical events can be tracked theoretically. Patients with DM visit different physicians due to multiple comorbidities and complications, which leads to loss of follow-up. Therefore, the use of this database would strengthen the acquisition of robust results.

This study has several limitations. In this study, we conducted multivariable Cox regression analyses. However, the possibility of unmeasured confounders and residual bias could not be eliminated. For instance, the information on the duration of DM is not available in the JMDC Claims Database. Similarly, data on socioeconomic status, which could affect the risk for CVD events, were not available in this dataset. Given that the JMDC Claims Database is an administrative claims database including individuals under the coverage of “kenpo” which is a health insurance system for employees, and most individuals enrolled in this database are employees (or their family members) of relatively large Japanese companies, the socioeconomic status of individuals in this database is not significantly different. Nevertheless, the lack of data on socioeconomic status should be considered a study limitation in this study. The JMDC Claims Database does not include people aged > 75 years; therefore, further investigation is required to validate our results in elderly patients with DM. As aforementioned, the present study focused on the primary prevention setting and the number of CVD events was not so large. Therefore, it would be difficult to generalize our results to other populations at high CVD risk. Using ICD-10 codes for the CVD diagnosis should be considered a major study limitation. In a real-world clinical setting, physicians may register certain disease names (using ICD-10 codes) only for reimbursement, which would overestimate the incidence of CVD. If echocardiography was performed in an individual with possible HF, most physicians in Japan register “suspected HF”. Therefore, we excluded individuals whose disease code with “suspect” to ensure validity. In addition, the accuracy of diagnostic codes in Japanese administrative data is reported to be high [33, 34], and the incidence of CVD events in the JMDC Claims Database is comparable to that in other epidemiological data in Japan [35, 36]. Hence, we believe that the potential overestimation of CVD diagnosis would not affect the primary results of our study so largely. However, the diagnoses recorded in administrative claims datasets (such as our dataset) should generally be considered less well validated, and uncertainty remains regarding the accuracy of the CVD diagnosis. Although all-cause mortality was comparable between the individual SGLT2 inhibitors, the JMDC Claims Database does not include data on cardiovascular death. Further investigations with a longer follow-up period are needed to confirm our results because a mean follow-up period was relatively short in this study.

## Conclusion

Our analysis of a nationwide real-world dataset suggested that the risk of cardiovascular events including HF, MI, AP, stroke, and AF would be comparable between individual SGLT2 inhibitors.

## Supplementary Information

**Additional file 1:** **Figure S1.** Study design. **Figure S2.** Risk of cardiovascular event among SGLT2 Inhibitors (continuous prescription group). **Figure S3.** Risk of cardiovascular event among SGLT2 inhibitors (induction period ≥ 90 days). **Figure S4.** Risk of cardiovascular event among SGLT2 inhibitors (multiple imputations for missing data). **Figure S5.** Risk of cardiovascular event among SGLT2 inhibitors (competing risks model). **Figure S6.** All-cause mortality among SGLT2 inhibitors. **Figure S7.** Risk of cardiovascular event among SGLT2 inhibitors (adjusted for estimated glomerular filtration rate). **Figure S8.** Risk of cardiovascular event among SGLT2 inhibitors (individuals with diagnosis of type 2 diabetes mellitus). **Figure S9.** Risk of cardiovascular event among SGLT2 inhibitors (comparison of six SGLT2 inhibitors).

## Data Availability

The JMDC Claims Database is available for anyone who purchases it from the JMDC Inc (https://www.jmdc.co.jp/en/index).

## Abbreviations

AF: Atrial fibrillation
AP: Angina pectoris
BMI: Body mass index
CI: Confidence interval
CVD: Cardiovascular disease
DM: Diabetes mellitus
HF: Heart failure
HR: Hazard ratio
MI: Myocardial infarction
SGLT2: Sodium-glucose cotransporter-2
