# Supplementary material for: Comparison of cardiovascular outcomes between SGLT2 inhibitors in diabetes mellitus
Source: Cardiovasc Diabetol. 2022 May 18;21:67. doi: 10.1186/s12933-022-01508-6 (PMC9115977; doi:10.1186/s12933-022-01508-6)
Supplement: Supplementary file 1 — Additional file 1: Figure S1. Study design. Figure S2. Risk of cardiovascular event among SGLT2 Inhibitors (continuous prescription group). Figure S3. Risk of cardiovascular event among SGLT2 inhibitors (induction period ≥ 90 days). Figure S4. Risk of cardiovascular event among SGLT2 inhibitors (multiple imputations for missing data). Figure S5. Risk of cardiovascular event among SGLT2 inhibitors (competing risks model). Figure S6. All-cause mortality among SGLT2 inhibitors. Figure S7. Risk of cardiovascular event among SGLT2 inhibitors (adjusted for estimated glomerular filtration rate). Figure S8. Risk of cardiovascular event among SGLT2 inhibitors (individuals with diagnosis of type 2 diabetes mellitus). Figure S9. Risk of cardiovascular event among SGLT2 inhibitors (comparison of six SGLT2 inhibitors). [file 12933_2022_1508_MOESM1_ESM.pptx]

## Slide 1
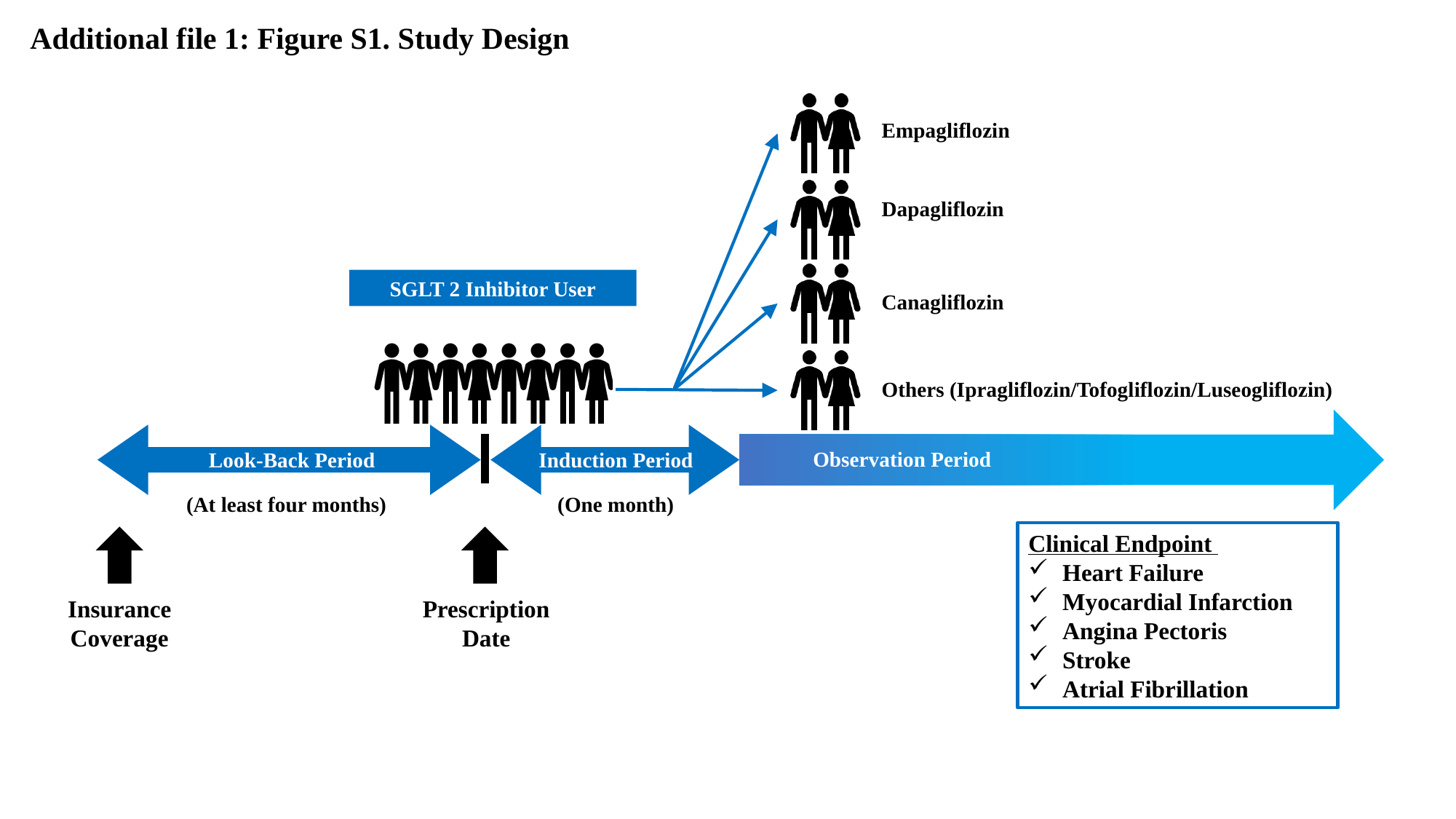

Additional file 1: Figure S1. Study Design
Empagliflozin
Dapagliflozin
SGLT 2 Inhibitor User
Canagliflozin
Others (Ipragliflozin/Tofogliflozin/Luseogliflozin)
Observation Period
Look-Back Period
Induction Period
(At least four months)
(One month)
Clinical Endpoint
Heart Failure
Myocardial Infarction
Angina Pectoris
Stroke
Atrial Fibrillation
Prescription
Date
Insurance
Coverage

## Slide 2
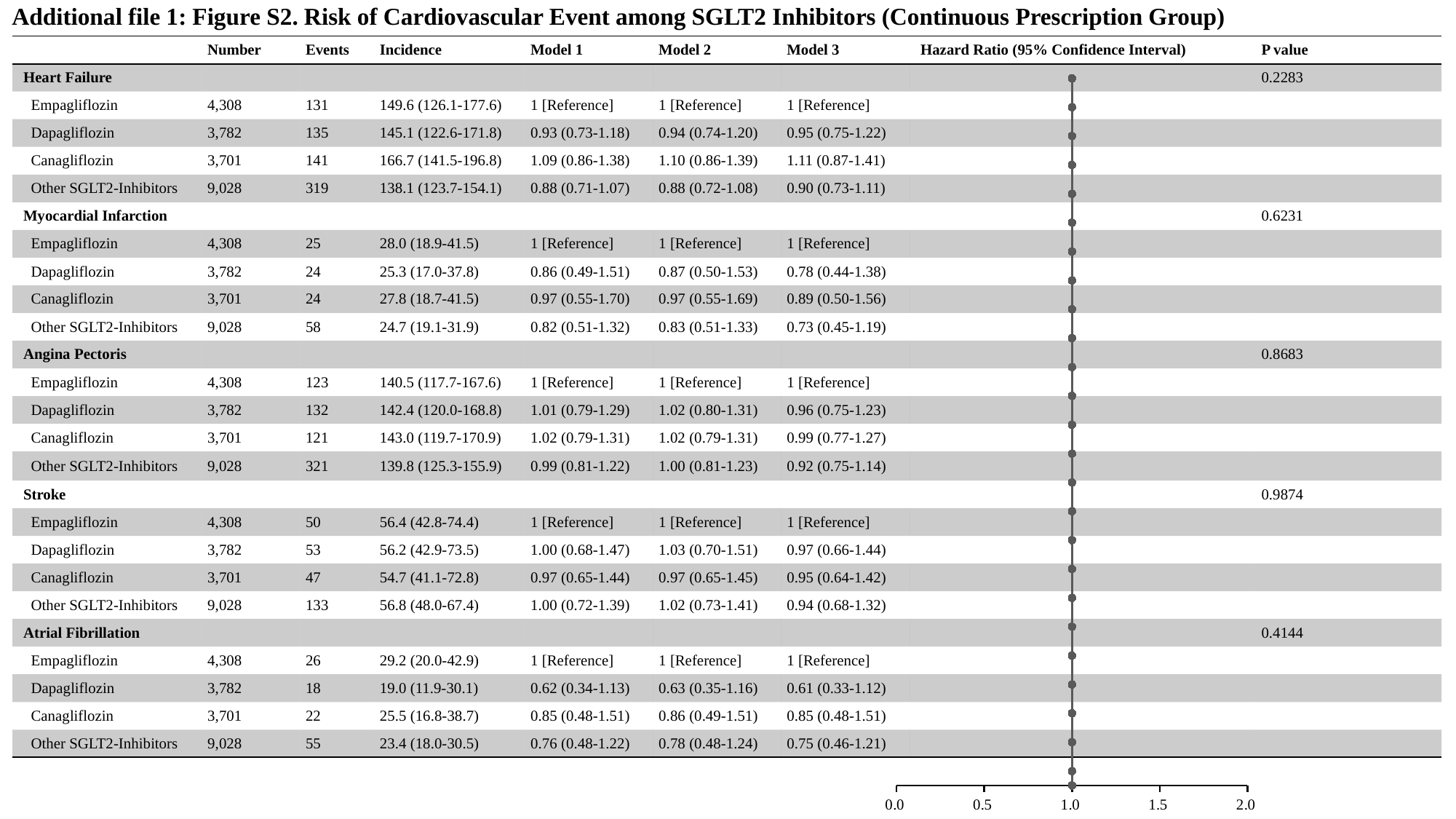

Additional file 1: Figure S2. Risk of Cardiovascular Event among SGLT2 Inhibitors (Continuous Prescription Group)
| | Number | Events | Incidence | Model 1 | Model 2 | Model 3 | Hazard Ratio (95% Confidence Interval) | P value |
| --- | --- | --- | --- | --- | --- | --- | --- | --- |
| Heart Failure | | | | | | | | 0.2283 |
| Empagliflozin | 4,308 | 131 | 149.6 (126.1-177.6) | 1 [Reference] | 1 [Reference] | 1 [Reference] | | |
| Dapagliflozin | 3,782 | 135 | 145.1 (122.6-171.8) | 0.93 (0.73-1.18) | 0.94 (0.74-1.20) | 0.95 (0.75-1.22) | | |
| Canagliflozin | 3,701 | 141 | 166.7 (141.5-196.8) | 1.09 (0.86-1.38) | 1.10 (0.86-1.39) | 1.11 (0.87-1.41) | | |
| Other SGLT2-Inhibitors | 9,028 | 319 | 138.1 (123.7-154.1) | 0.88 (0.71-1.07) | 0.88 (0.72-1.08) | 0.90 (0.73-1.11) | | |
| Myocardial Infarction | | | | | | | | 0.6231 |
| Empagliflozin | 4,308 | 25 | 28.0 (18.9-41.5) | 1 [Reference] | 1 [Reference] | 1 [Reference] | | |
| Dapagliflozin | 3,782 | 24 | 25.3 (17.0-37.8) | 0.86 (0.49-1.51) | 0.87 (0.50-1.53) | 0.78 (0.44-1.38) | | |
| Canagliflozin | 3,701 | 24 | 27.8 (18.7-41.5) | 0.97 (0.55-1.70) | 0.97 (0.55-1.69) | 0.89 (0.50-1.56) | | |
| Other SGLT2-Inhibitors | 9,028 | 58 | 24.7 (19.1-31.9) | 0.82 (0.51-1.32) | 0.83 (0.51-1.33) | 0.73 (0.45-1.19) | | |
| Angina Pectoris | | | | | | | | 0.8683 |
| Empagliflozin | 4,308 | 123 | 140.5 (117.7-167.6) | 1 [Reference] | 1 [Reference] | 1 [Reference] | | |
| Dapagliflozin | 3,782 | 132 | 142.4 (120.0-168.8) | 1.01 (0.79-1.29) | 1.02 (0.80-1.31) | 0.96 (0.75-1.23) | | |
| Canagliflozin | 3,701 | 121 | 143.0 (119.7-170.9) | 1.02 (0.79-1.31) | 1.02 (0.79-1.31) | 0.99 (0.77-1.27) | | |
| Other SGLT2-Inhibitors | 9,028 | 321 | 139.8 (125.3-155.9) | 0.99 (0.81-1.22) | 1.00 (0.81-1.23) | 0.92 (0.75-1.14) | | |
| Stroke | | | | | | | | 0.9874 |
| Empagliflozin | 4,308 | 50 | 56.4 (42.8-74.4) | 1 [Reference] | 1 [Reference] | 1 [Reference] | | |
| Dapagliflozin | 3,782 | 53 | 56.2 (42.9-73.5) | 1.00 (0.68-1.47) | 1.03 (0.70-1.51) | 0.97 (0.66-1.44) | | |
| Canagliflozin | 3,701 | 47 | 54.7 (41.1-72.8) | 0.97 (0.65-1.44) | 0.97 (0.65-1.45) | 0.95 (0.64-1.42) | | |
| Other SGLT2-Inhibitors | 9,028 | 133 | 56.8 (48.0-67.4) | 1.00 (0.72-1.39) | 1.02 (0.73-1.41) | 0.94 (0.68-1.32) | | |
| Atrial Fibrillation | | | | | | | | 0.4144 |
| Empagliflozin | 4,308 | 26 | 29.2 (20.0-42.9) | 1 [Reference] | 1 [Reference] | 1 [Reference] | | |
| Dapagliflozin | 3,782 | 18 | 19.0 (11.9-30.1) | 0.62 (0.34-1.13) | 0.63 (0.35-1.16) | 0.61 (0.33-1.12) | | |
| Canagliflozin | 3,701 | 22 | 25.5 (16.8-38.7) | 0.85 (0.48-1.51) | 0.86 (0.49-1.51) | 0.85 (0.48-1.51) | | |
| Other SGLT2-Inhibitors | 9,028 | 55 | 23.4 (18.0-30.5) | 0.76 (0.48-1.22) | 0.78 (0.48-1.24) | 0.75 (0.46-1.21) | | |
### Chart
| Category | | | | |
|---|---|---|---|---|

## Slide 3
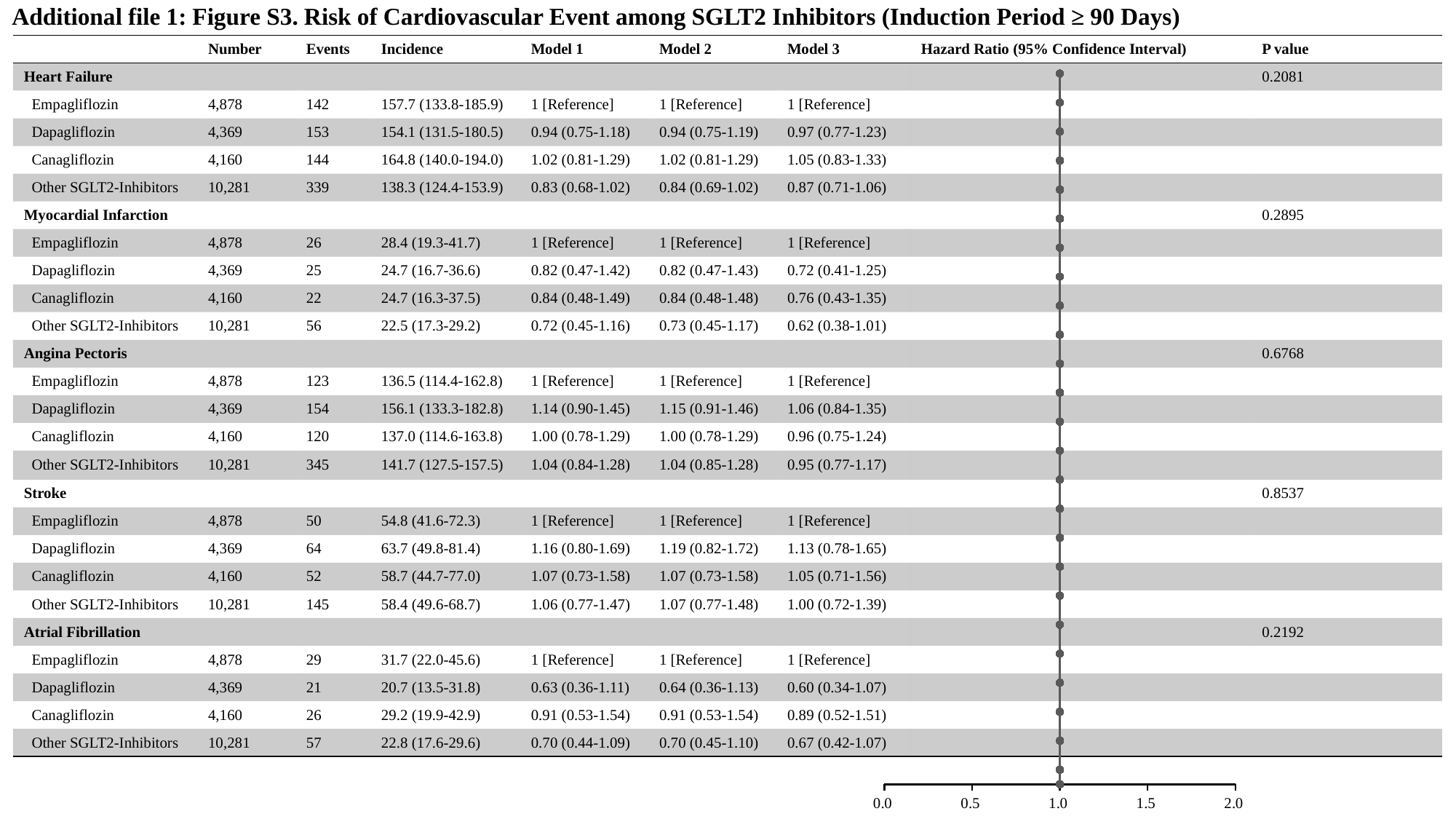

Additional file 1: Figure S3. Risk of Cardiovascular Event among SGLT2 Inhibitors (Induction Period ≥ 90 Days)
| | Number | Events | Incidence | Model 1 | Model 2 | Model 3 | Hazard Ratio (95% Confidence Interval) | P value |
| --- | --- | --- | --- | --- | --- | --- | --- | --- |
| Heart Failure | | | | | | | | 0.2081 |
| Empagliflozin | 4,878 | 142 | 157.7 (133.8-185.9) | 1 [Reference] | 1 [Reference] | 1 [Reference] | | |
| Dapagliflozin | 4,369 | 153 | 154.1 (131.5-180.5) | 0.94 (0.75-1.18) | 0.94 (0.75-1.19) | 0.97 (0.77-1.23) | | |
| Canagliflozin | 4,160 | 144 | 164.8 (140.0-194.0) | 1.02 (0.81-1.29) | 1.02 (0.81-1.29) | 1.05 (0.83-1.33) | | |
| Other SGLT2-Inhibitors | 10,281 | 339 | 138.3 (124.4-153.9) | 0.83 (0.68-1.02) | 0.84 (0.69-1.02) | 0.87 (0.71-1.06) | | |
| Myocardial Infarction | | | | | | | | 0.2895 |
| Empagliflozin | 4,878 | 26 | 28.4 (19.3-41.7) | 1 [Reference] | 1 [Reference] | 1 [Reference] | | |
| Dapagliflozin | 4,369 | 25 | 24.7 (16.7-36.6) | 0.82 (0.47-1.42) | 0.82 (0.47-1.43) | 0.72 (0.41-1.25) | | |
| Canagliflozin | 4,160 | 22 | 24.7 (16.3-37.5) | 0.84 (0.48-1.49) | 0.84 (0.48-1.48) | 0.76 (0.43-1.35) | | |
| Other SGLT2-Inhibitors | 10,281 | 56 | 22.5 (17.3-29.2) | 0.72 (0.45-1.16) | 0.73 (0.45-1.17) | 0.62 (0.38-1.01) | | |
| Angina Pectoris | | | | | | | | 0.6768 |
| Empagliflozin | 4,878 | 123 | 136.5 (114.4-162.8) | 1 [Reference] | 1 [Reference] | 1 [Reference] | | |
| Dapagliflozin | 4,369 | 154 | 156.1 (133.3-182.8) | 1.14 (0.90-1.45) | 1.15 (0.91-1.46) | 1.06 (0.84-1.35) | | |
| Canagliflozin | 4,160 | 120 | 137.0 (114.6-163.8) | 1.00 (0.78-1.29) | 1.00 (0.78-1.29) | 0.96 (0.75-1.24) | | |
| Other SGLT2-Inhibitors | 10,281 | 345 | 141.7 (127.5-157.5) | 1.04 (0.84-1.28) | 1.04 (0.85-1.28) | 0.95 (0.77-1.17) | | |
| Stroke | | | | | | | | 0.8537 |
| Empagliflozin | 4,878 | 50 | 54.8 (41.6-72.3) | 1 [Reference] | 1 [Reference] | 1 [Reference] | | |
| Dapagliflozin | 4,369 | 64 | 63.7 (49.8-81.4) | 1.16 (0.80-1.69) | 1.19 (0.82-1.72) | 1.13 (0.78-1.65) | | |
| Canagliflozin | 4,160 | 52 | 58.7 (44.7-77.0) | 1.07 (0.73-1.58) | 1.07 (0.73-1.58) | 1.05 (0.71-1.56) | | |
| Other SGLT2-Inhibitors | 10,281 | 145 | 58.4 (49.6-68.7) | 1.06 (0.77-1.47) | 1.07 (0.77-1.48) | 1.00 (0.72-1.39) | | |
| Atrial Fibrillation | | | | | | | | 0.2192 |
| Empagliflozin | 4,878 | 29 | 31.7 (22.0-45.6) | 1 [Reference] | 1 [Reference] | 1 [Reference] | | |
| Dapagliflozin | 4,369 | 21 | 20.7 (13.5-31.8) | 0.63 (0.36-1.11) | 0.64 (0.36-1.13) | 0.60 (0.34-1.07) | | |
| Canagliflozin | 4,160 | 26 | 29.2 (19.9-42.9) | 0.91 (0.53-1.54) | 0.91 (0.53-1.54) | 0.89 (0.52-1.51) | | |
| Other SGLT2-Inhibitors | 10,281 | 57 | 22.8 (17.6-29.6) | 0.70 (0.44-1.09) | 0.70 (0.45-1.10) | 0.67 (0.42-1.07) | | |
### Chart
| Category | | | | |
|---|---|---|---|---|

## Slide 4
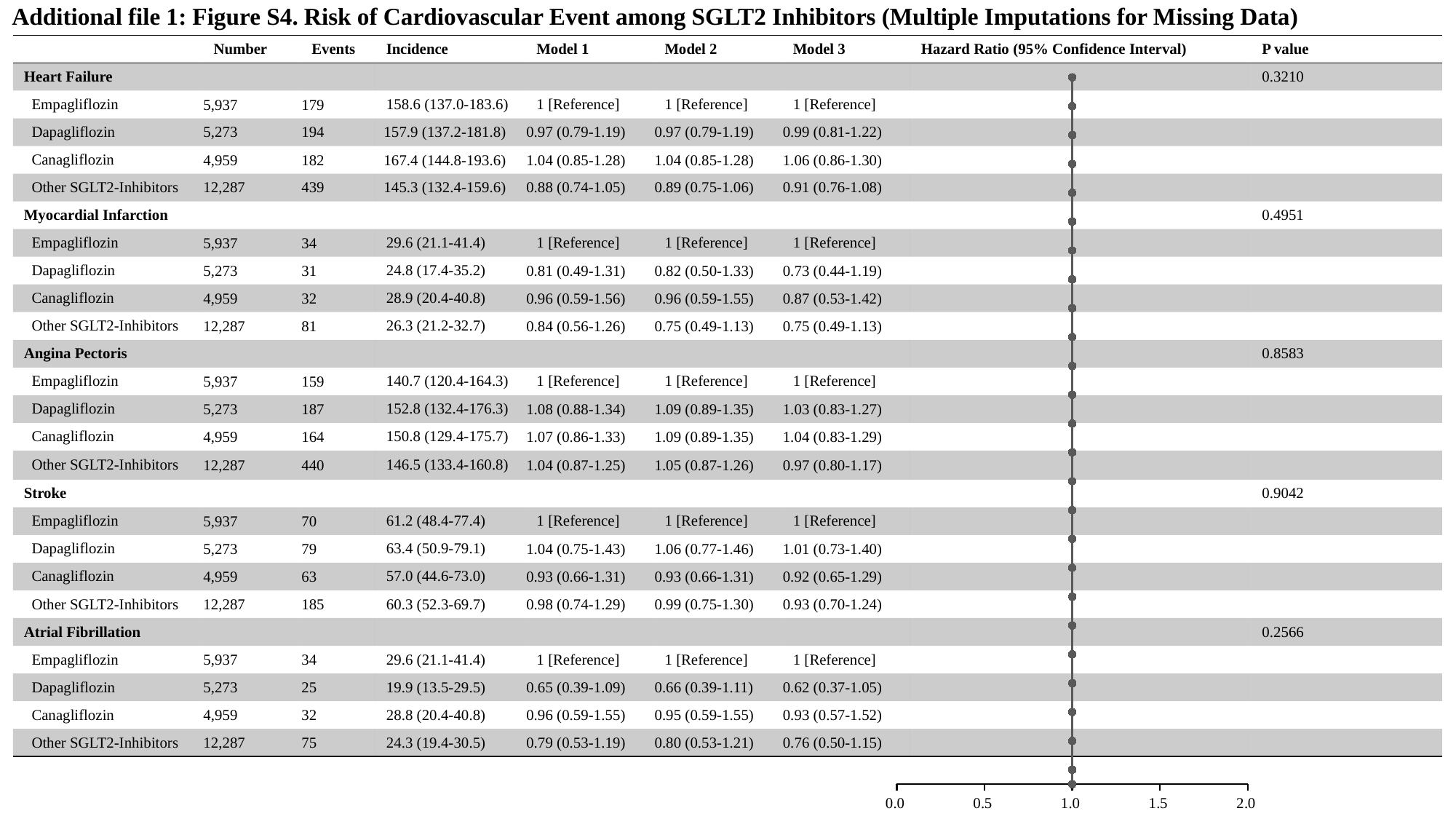

Additional file 1: Figure S4. Risk of Cardiovascular Event among SGLT2 Inhibitors (Multiple Imputations for Missing Data)
| | Number | Events | Incidence | Model 1 | Model 2 | Model 3 | Hazard Ratio (95% Confidence Interval) | P value |
| --- | --- | --- | --- | --- | --- | --- | --- | --- |
| Heart Failure | | | | | | | | 0.3210 |
| Empagliflozin | 5,937 | 179 | 158.6 (137.0-183.6) | 1 [Reference] | 1 [Reference] | 1 [Reference] | | |
| Dapagliflozin | 5,273 | 194 | 157.9 (137.2-181.8) | 0.97 (0.79-1.19) | 0.97 (0.79-1.19) | 0.99 (0.81-1.22) | | |
| Canagliflozin | 4,959 | 182 | 167.4 (144.8-193.6) | 1.04 (0.85-1.28) | 1.04 (0.85-1.28) | 1.06 (0.86-1.30) | | |
| Other SGLT2-Inhibitors | 12,287 | 439 | 145.3 (132.4-159.6) | 0.88 (0.74-1.05) | 0.89 (0.75-1.06) | 0.91 (0.76-1.08) | | |
| Myocardial Infarction | | | | | | | | 0.4951 |
| Empagliflozin | 5,937 | 34 | 29.6 (21.1-41.4) | 1 [Reference] | 1 [Reference] | 1 [Reference] | | |
| Dapagliflozin | 5,273 | 31 | 24.8 (17.4-35.2) | 0.81 (0.49-1.31) | 0.82 (0.50-1.33) | 0.73 (0.44-1.19) | | |
| Canagliflozin | 4,959 | 32 | 28.9 (20.4-40.8) | 0.96 (0.59-1.56) | 0.96 (0.59-1.55) | 0.87 (0.53-1.42) | | |
| Other SGLT2-Inhibitors | 12,287 | 81 | 26.3 (21.2-32.7) | 0.84 (0.56-1.26) | 0.75 (0.49-1.13) | 0.75 (0.49-1.13) | | |
| Angina Pectoris | | | | | | | | 0.8583 |
| Empagliflozin | 5,937 | 159 | 140.7 (120.4-164.3) | 1 [Reference] | 1 [Reference] | 1 [Reference] | | |
| Dapagliflozin | 5,273 | 187 | 152.8 (132.4-176.3) | 1.08 (0.88-1.34) | 1.09 (0.89-1.35) | 1.03 (0.83-1.27) | | |
| Canagliflozin | 4,959 | 164 | 150.8 (129.4-175.7) | 1.07 (0.86-1.33) | 1.09 (0.89-1.35) | 1.04 (0.83-1.29) | | |
| Other SGLT2-Inhibitors | 12,287 | 440 | 146.5 (133.4-160.8) | 1.04 (0.87-1.25) | 1.05 (0.87-1.26) | 0.97 (0.80-1.17) | | |
| Stroke | | | | | | | | 0.9042 |
| Empagliflozin | 5,937 | 70 | 61.2 (48.4-77.4) | 1 [Reference] | 1 [Reference] | 1 [Reference] | | |
| Dapagliflozin | 5,273 | 79 | 63.4 (50.9-79.1) | 1.04 (0.75-1.43) | 1.06 (0.77-1.46) | 1.01 (0.73-1.40) | | |
| Canagliflozin | 4,959 | 63 | 57.0 (44.6-73.0) | 0.93 (0.66-1.31) | 0.93 (0.66-1.31) | 0.92 (0.65-1.29) | | |
| Other SGLT2-Inhibitors | 12,287 | 185 | 60.3 (52.3-69.7) | 0.98 (0.74-1.29) | 0.99 (0.75-1.30) | 0.93 (0.70-1.24) | | |
| Atrial Fibrillation | | | | | | | | 0.2566 |
| Empagliflozin | 5,937 | 34 | 29.6 (21.1-41.4) | 1 [Reference] | 1 [Reference] | 1 [Reference] | | |
| Dapagliflozin | 5,273 | 25 | 19.9 (13.5-29.5) | 0.65 (0.39-1.09) | 0.66 (0.39-1.11) | 0.62 (0.37-1.05) | | |
| Canagliflozin | 4,959 | 32 | 28.8 (20.4-40.8) | 0.96 (0.59-1.55) | 0.95 (0.59-1.55) | 0.93 (0.57-1.52) | | |
| Other SGLT2-Inhibitors | 12,287 | 75 | 24.3 (19.4-30.5) | 0.79 (0.53-1.19) | 0.80 (0.53-1.21) | 0.76 (0.50-1.15) | | |
### Chart
| Category | | | | |
|---|---|---|---|---|

## Slide 5
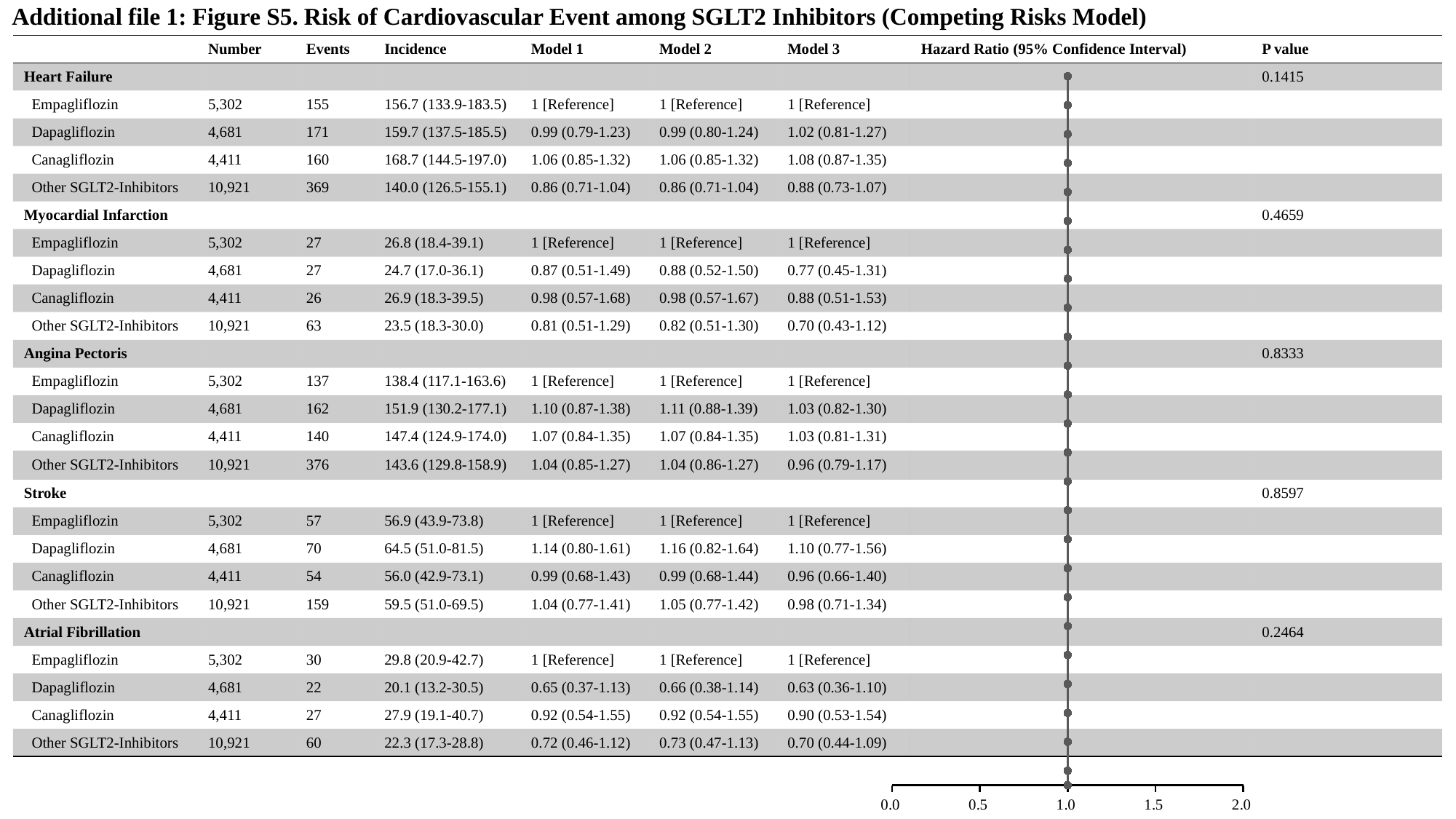

Additional file 1: Figure S5. Risk of Cardiovascular Event among SGLT2 Inhibitors (Competing Risks Model)
| | Number | Events | Incidence | Model 1 | Model 2 | Model 3 | Hazard Ratio (95% Confidence Interval) | P value |
| --- | --- | --- | --- | --- | --- | --- | --- | --- |
| Heart Failure | | | | | | | | 0.1415 |
| Empagliflozin | 5,302 | 155 | 156.7 (133.9-183.5) | 1 [Reference] | 1 [Reference] | 1 [Reference] | | |
| Dapagliflozin | 4,681 | 171 | 159.7 (137.5-185.5) | 0.99 (0.79-1.23) | 0.99 (0.80-1.24) | 1.02 (0.81-1.27) | | |
| Canagliflozin | 4,411 | 160 | 168.7 (144.5-197.0) | 1.06 (0.85-1.32) | 1.06 (0.85-1.32) | 1.08 (0.87-1.35) | | |
| Other SGLT2-Inhibitors | 10,921 | 369 | 140.0 (126.5-155.1) | 0.86 (0.71-1.04) | 0.86 (0.71-1.04) | 0.88 (0.73-1.07) | | |
| Myocardial Infarction | | | | | | | | 0.4659 |
| Empagliflozin | 5,302 | 27 | 26.8 (18.4-39.1) | 1 [Reference] | 1 [Reference] | 1 [Reference] | | |
| Dapagliflozin | 4,681 | 27 | 24.7 (17.0-36.1) | 0.87 (0.51-1.49) | 0.88 (0.52-1.50) | 0.77 (0.45-1.31) | | |
| Canagliflozin | 4,411 | 26 | 26.9 (18.3-39.5) | 0.98 (0.57-1.68) | 0.98 (0.57-1.67) | 0.88 (0.51-1.53) | | |
| Other SGLT2-Inhibitors | 10,921 | 63 | 23.5 (18.3-30.0) | 0.81 (0.51-1.29) | 0.82 (0.51-1.30) | 0.70 (0.43-1.12) | | |
| Angina Pectoris | | | | | | | | 0.8333 |
| Empagliflozin | 5,302 | 137 | 138.4 (117.1-163.6) | 1 [Reference] | 1 [Reference] | 1 [Reference] | | |
| Dapagliflozin | 4,681 | 162 | 151.9 (130.2-177.1) | 1.10 (0.87-1.38) | 1.11 (0.88-1.39) | 1.03 (0.82-1.30) | | |
| Canagliflozin | 4,411 | 140 | 147.4 (124.9-174.0) | 1.07 (0.84-1.35) | 1.07 (0.84-1.35) | 1.03 (0.81-1.31) | | |
| Other SGLT2-Inhibitors | 10,921 | 376 | 143.6 (129.8-158.9) | 1.04 (0.85-1.27) | 1.04 (0.86-1.27) | 0.96 (0.79-1.17) | | |
| Stroke | | | | | | | | 0.8597 |
| Empagliflozin | 5,302 | 57 | 56.9 (43.9-73.8) | 1 [Reference] | 1 [Reference] | 1 [Reference] | | |
| Dapagliflozin | 4,681 | 70 | 64.5 (51.0-81.5) | 1.14 (0.80-1.61) | 1.16 (0.82-1.64) | 1.10 (0.77-1.56) | | |
| Canagliflozin | 4,411 | 54 | 56.0 (42.9-73.1) | 0.99 (0.68-1.43) | 0.99 (0.68-1.44) | 0.96 (0.66-1.40) | | |
| Other SGLT2-Inhibitors | 10,921 | 159 | 59.5 (51.0-69.5) | 1.04 (0.77-1.41) | 1.05 (0.77-1.42) | 0.98 (0.71-1.34) | | |
| Atrial Fibrillation | | | | | | | | 0.2464 |
| Empagliflozin | 5,302 | 30 | 29.8 (20.9-42.7) | 1 [Reference] | 1 [Reference] | 1 [Reference] | | |
| Dapagliflozin | 4,681 | 22 | 20.1 (13.2-30.5) | 0.65 (0.37-1.13) | 0.66 (0.38-1.14) | 0.63 (0.36-1.10) | | |
| Canagliflozin | 4,411 | 27 | 27.9 (19.1-40.7) | 0.92 (0.54-1.55) | 0.92 (0.54-1.55) | 0.90 (0.53-1.54) | | |
| Other SGLT2-Inhibitors | 10,921 | 60 | 22.3 (17.3-28.8) | 0.72 (0.46-1.12) | 0.73 (0.47-1.13) | 0.70 (0.44-1.09) | | |
### Chart
| Category | | | | |
|---|---|---|---|---|

## Slide 6
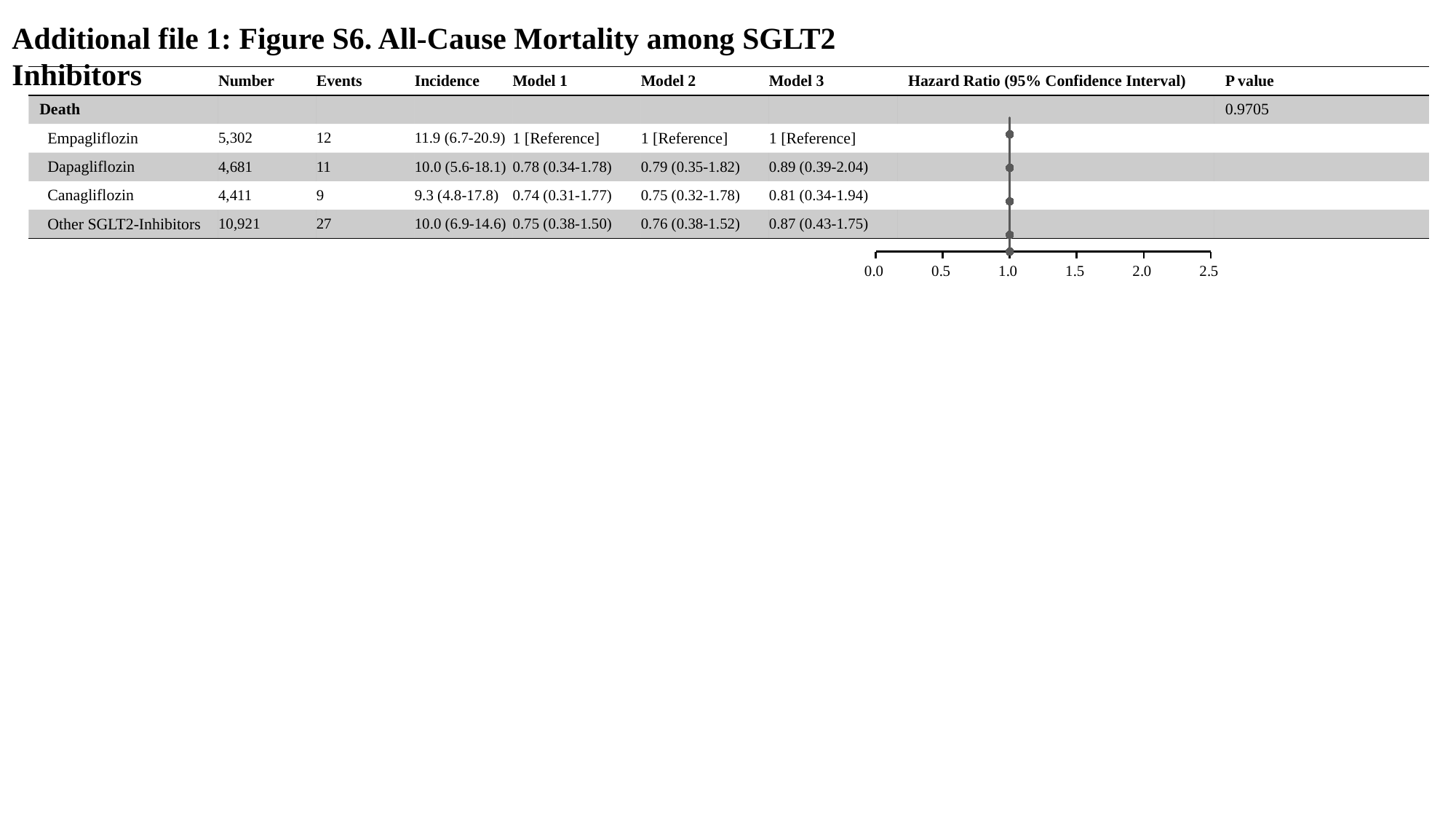

Additional file 1: Figure S6. All-Cause Mortality among SGLT2 Inhibitors
| | Number | Events | Incidence | Model 1 | Model 2 | Model 3 | Hazard Ratio (95% Confidence Interval) | P value |
| --- | --- | --- | --- | --- | --- | --- | --- | --- |
| Death | | | | | | | | 0.9705 |
| Empagliflozin | 5,302 | 12 | 11.9 (6.7-20.9) | 1 [Reference] | 1 [Reference] | 1 [Reference] | | |
| Dapagliflozin | 4,681 | 11 | 10.0 (5.6-18.1) | 0.78 (0.34-1.78) | 0.79 (0.35-1.82) | 0.89 (0.39-2.04) | | |
| Canagliflozin | 4,411 | 9 | 9.3 (4.8-17.8) | 0.74 (0.31-1.77) | 0.75 (0.32-1.78) | 0.81 (0.34-1.94) | | |
| Other SGLT2-Inhibitors | 10,921 | 27 | 10.0 (6.9-14.6) | 0.75 (0.38-1.50) | 0.76 (0.38-1.52) | 0.87 (0.43-1.75) | | |
### Chart
| Category | | | | |
|---|---|---|---|---|

## Slide 7
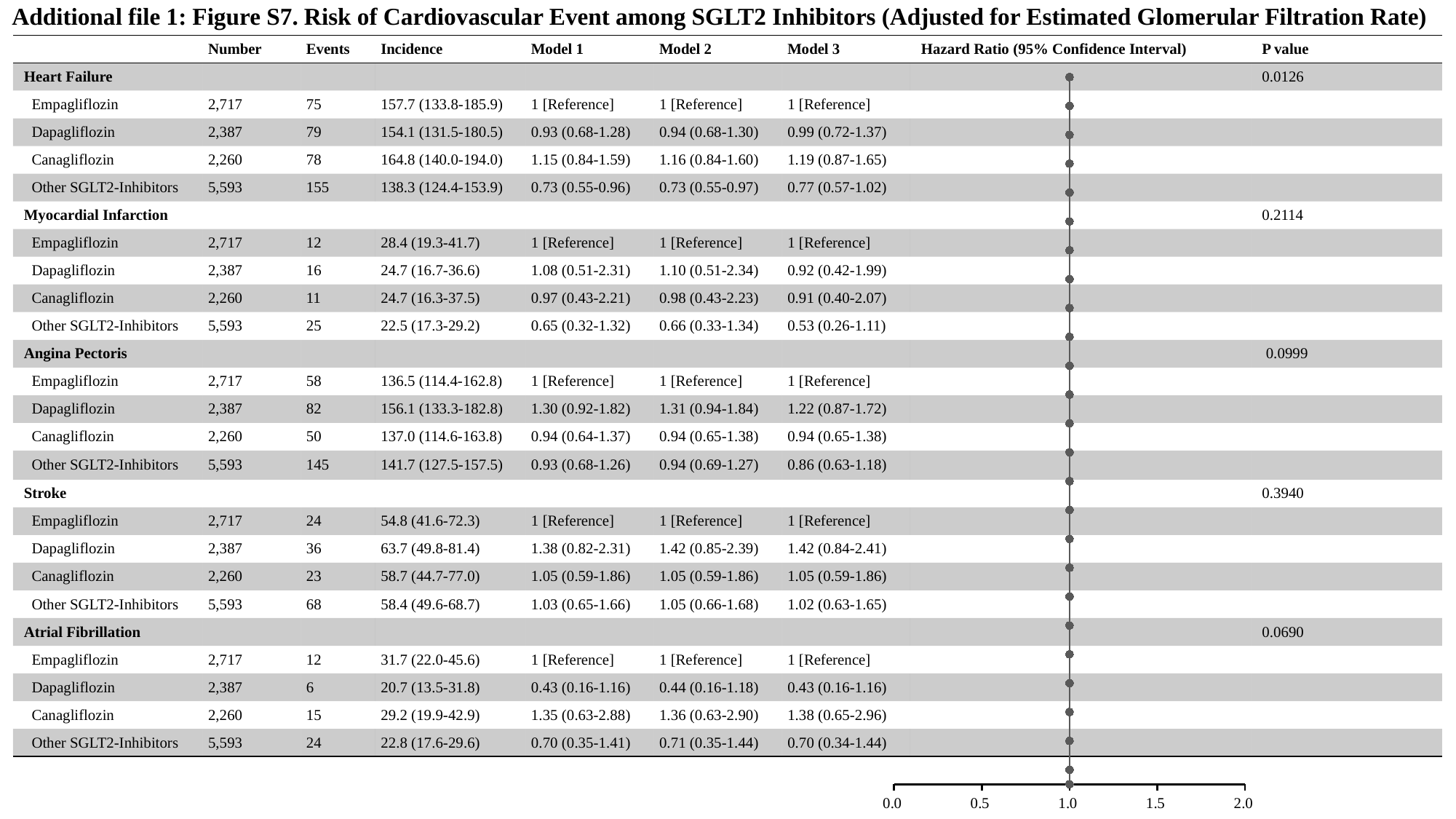

Additional file 1: Figure S7. Risk of Cardiovascular Event among SGLT2 Inhibitors (Adjusted for Estimated Glomerular Filtration Rate)
| | Number | Events | Incidence | Model 1 | Model 2 | Model 3 | Hazard Ratio (95% Confidence Interval) | P value |
| --- | --- | --- | --- | --- | --- | --- | --- | --- |
| Heart Failure | | | | | | | | 0.0126 |
| Empagliflozin | 2,717 | 75 | 157.7 (133.8-185.9) | 1 [Reference] | 1 [Reference] | 1 [Reference] | | |
| Dapagliflozin | 2,387 | 79 | 154.1 (131.5-180.5) | 0.93 (0.68-1.28) | 0.94 (0.68-1.30) | 0.99 (0.72-1.37) | | |
| Canagliflozin | 2,260 | 78 | 164.8 (140.0-194.0) | 1.15 (0.84-1.59) | 1.16 (0.84-1.60) | 1.19 (0.87-1.65) | | |
| Other SGLT2-Inhibitors | 5,593 | 155 | 138.3 (124.4-153.9) | 0.73 (0.55-0.96) | 0.73 (0.55-0.97) | 0.77 (0.57-1.02) | | |
| Myocardial Infarction | | | | | | | | 0.2114 |
| Empagliflozin | 2,717 | 12 | 28.4 (19.3-41.7) | 1 [Reference] | 1 [Reference] | 1 [Reference] | | |
| Dapagliflozin | 2,387 | 16 | 24.7 (16.7-36.6) | 1.08 (0.51-2.31) | 1.10 (0.51-2.34) | 0.92 (0.42-1.99) | | |
| Canagliflozin | 2,260 | 11 | 24.7 (16.3-37.5) | 0.97 (0.43-2.21) | 0.98 (0.43-2.23) | 0.91 (0.40-2.07) | | |
| Other SGLT2-Inhibitors | 5,593 | 25 | 22.5 (17.3-29.2) | 0.65 (0.32-1.32) | 0.66 (0.33-1.34) | 0.53 (0.26-1.11) | | |
| Angina Pectoris | | | | | | | | 0.0999 |
| Empagliflozin | 2,717 | 58 | 136.5 (114.4-162.8) | 1 [Reference] | 1 [Reference] | 1 [Reference] | | |
| Dapagliflozin | 2,387 | 82 | 156.1 (133.3-182.8) | 1.30 (0.92-1.82) | 1.31 (0.94-1.84) | 1.22 (0.87-1.72) | | |
| Canagliflozin | 2,260 | 50 | 137.0 (114.6-163.8) | 0.94 (0.64-1.37) | 0.94 (0.65-1.38) | 0.94 (0.65-1.38) | | |
| Other SGLT2-Inhibitors | 5,593 | 145 | 141.7 (127.5-157.5) | 0.93 (0.68-1.26) | 0.94 (0.69-1.27) | 0.86 (0.63-1.18) | | |
| Stroke | | | | | | | | 0.3940 |
| Empagliflozin | 2,717 | 24 | 54.8 (41.6-72.3) | 1 [Reference] | 1 [Reference] | 1 [Reference] | | |
| Dapagliflozin | 2,387 | 36 | 63.7 (49.8-81.4) | 1.38 (0.82-2.31) | 1.42 (0.85-2.39) | 1.42 (0.84-2.41) | | |
| Canagliflozin | 2,260 | 23 | 58.7 (44.7-77.0) | 1.05 (0.59-1.86) | 1.05 (0.59-1.86) | 1.05 (0.59-1.86) | | |
| Other SGLT2-Inhibitors | 5,593 | 68 | 58.4 (49.6-68.7) | 1.03 (0.65-1.66) | 1.05 (0.66-1.68) | 1.02 (0.63-1.65) | | |
| Atrial Fibrillation | | | | | | | | 0.0690 |
| Empagliflozin | 2,717 | 12 | 31.7 (22.0-45.6) | 1 [Reference] | 1 [Reference] | 1 [Reference] | | |
| Dapagliflozin | 2,387 | 6 | 20.7 (13.5-31.8) | 0.43 (0.16-1.16) | 0.44 (0.16-1.18) | 0.43 (0.16-1.16) | | |
| Canagliflozin | 2,260 | 15 | 29.2 (19.9-42.9) | 1.35 (0.63-2.88) | 1.36 (0.63-2.90) | 1.38 (0.65-2.96) | | |
| Other SGLT2-Inhibitors | 5,593 | 24 | 22.8 (17.6-29.6) | 0.70 (0.35-1.41) | 0.71 (0.35-1.44) | 0.70 (0.34-1.44) | | |
### Chart
| Category | | | | |
|---|---|---|---|---|

## Slide 8
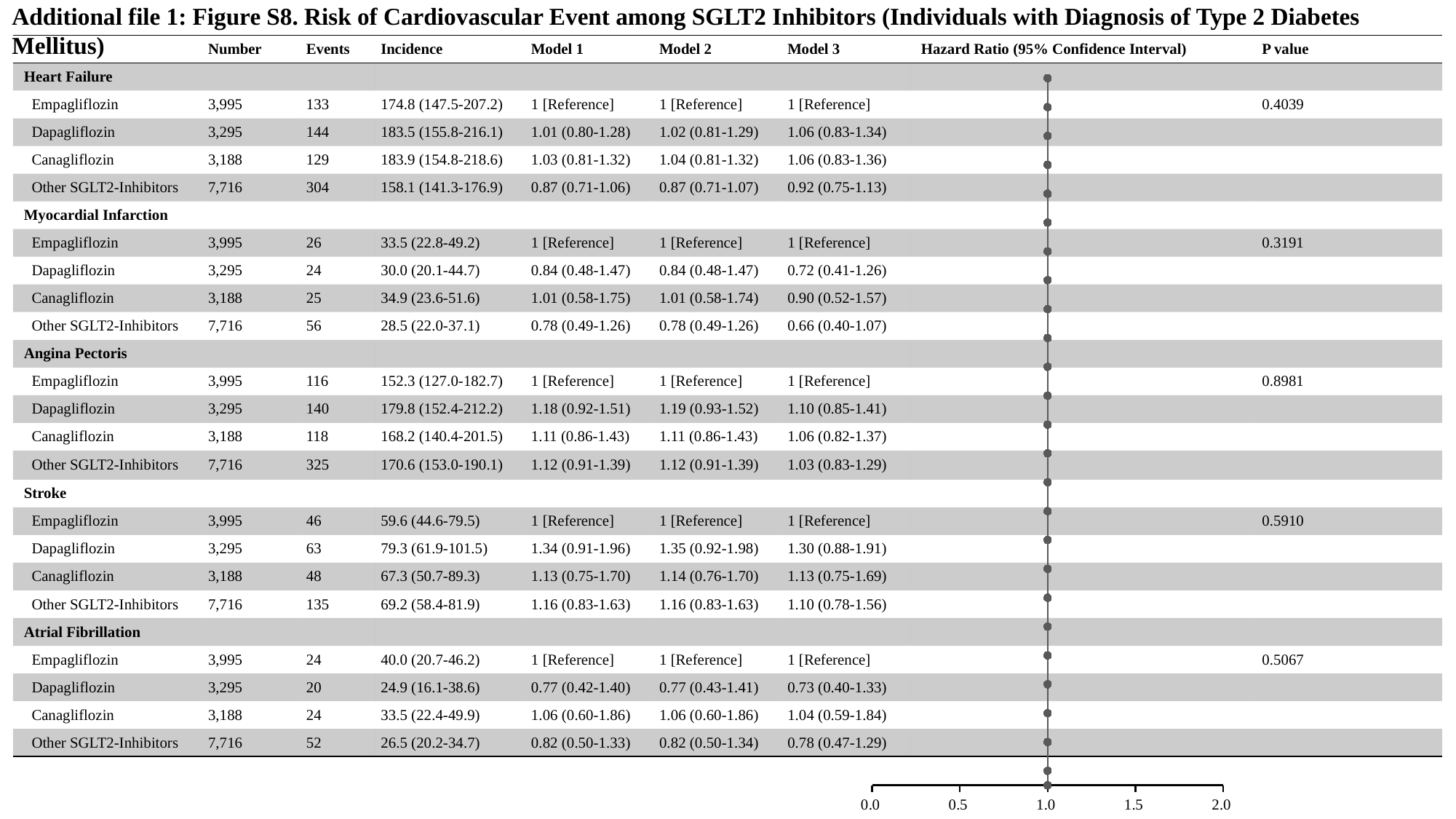

Additional file 1: Figure S8. Risk of Cardiovascular Event among SGLT2 Inhibitors (Individuals with Diagnosis of Type 2 Diabetes Mellitus)
| | Number | Events | Incidence | Model 1 | Model 2 | Model 3 | Hazard Ratio (95% Confidence Interval) | P value |
| --- | --- | --- | --- | --- | --- | --- | --- | --- |
| Heart Failure | | | | | | | | |
| Empagliflozin | 3,995 | 133 | 174.8 (147.5-207.2) | 1 [Reference] | 1 [Reference] | 1 [Reference] | | 0.4039 |
| Dapagliflozin | 3,295 | 144 | 183.5 (155.8-216.1) | 1.01 (0.80-1.28) | 1.02 (0.81-1.29) | 1.06 (0.83-1.34) | | |
| Canagliflozin | 3,188 | 129 | 183.9 (154.8-218.6) | 1.03 (0.81-1.32) | 1.04 (0.81-1.32) | 1.06 (0.83-1.36) | | |
| Other SGLT2-Inhibitors | 7,716 | 304 | 158.1 (141.3-176.9) | 0.87 (0.71-1.06) | 0.87 (0.71-1.07) | 0.92 (0.75-1.13) | | |
| Myocardial Infarction | | | | | | | | |
| Empagliflozin | 3,995 | 26 | 33.5 (22.8-49.2) | 1 [Reference] | 1 [Reference] | 1 [Reference] | | 0.3191 |
| Dapagliflozin | 3,295 | 24 | 30.0 (20.1-44.7) | 0.84 (0.48-1.47) | 0.84 (0.48-1.47) | 0.72 (0.41-1.26) | | |
| Canagliflozin | 3,188 | 25 | 34.9 (23.6-51.6) | 1.01 (0.58-1.75) | 1.01 (0.58-1.74) | 0.90 (0.52-1.57) | | |
| Other SGLT2-Inhibitors | 7,716 | 56 | 28.5 (22.0-37.1) | 0.78 (0.49-1.26) | 0.78 (0.49-1.26) | 0.66 (0.40-1.07) | | |
| Angina Pectoris | | | | | | | | |
| Empagliflozin | 3,995 | 116 | 152.3 (127.0-182.7) | 1 [Reference] | 1 [Reference] | 1 [Reference] | | 0.8981 |
| Dapagliflozin | 3,295 | 140 | 179.8 (152.4-212.2) | 1.18 (0.92-1.51) | 1.19 (0.93-1.52) | 1.10 (0.85-1.41) | | |
| Canagliflozin | 3,188 | 118 | 168.2 (140.4-201.5) | 1.11 (0.86-1.43) | 1.11 (0.86-1.43) | 1.06 (0.82-1.37) | | |
| Other SGLT2-Inhibitors | 7,716 | 325 | 170.6 (153.0-190.1) | 1.12 (0.91-1.39) | 1.12 (0.91-1.39) | 1.03 (0.83-1.29) | | |
| Stroke | | | | | | | | |
| Empagliflozin | 3,995 | 46 | 59.6 (44.6-79.5) | 1 [Reference] | 1 [Reference] | 1 [Reference] | | 0.5910 |
| Dapagliflozin | 3,295 | 63 | 79.3 (61.9-101.5) | 1.34 (0.91-1.96) | 1.35 (0.92-1.98) | 1.30 (0.88-1.91) | | |
| Canagliflozin | 3,188 | 48 | 67.3 (50.7-89.3) | 1.13 (0.75-1.70) | 1.14 (0.76-1.70) | 1.13 (0.75-1.69) | | |
| Other SGLT2-Inhibitors | 7,716 | 135 | 69.2 (58.4-81.9) | 1.16 (0.83-1.63) | 1.16 (0.83-1.63) | 1.10 (0.78-1.56) | | |
| Atrial Fibrillation | | | | | | | | |
| Empagliflozin | 3,995 | 24 | 40.0 (20.7-46.2) | 1 [Reference] | 1 [Reference] | 1 [Reference] | | 0.5067 |
| Dapagliflozin | 3,295 | 20 | 24.9 (16.1-38.6) | 0.77 (0.42-1.40) | 0.77 (0.43-1.41) | 0.73 (0.40-1.33) | | |
| Canagliflozin | 3,188 | 24 | 33.5 (22.4-49.9) | 1.06 (0.60-1.86) | 1.06 (0.60-1.86) | 1.04 (0.59-1.84) | | |
| Other SGLT2-Inhibitors | 7,716 | 52 | 26.5 (20.2-34.7) | 0.82 (0.50-1.33) | 0.82 (0.50-1.34) | 0.78 (0.47-1.29) | | |
### Chart
| Category | | | | |
|---|---|---|---|---|

## Slide 9
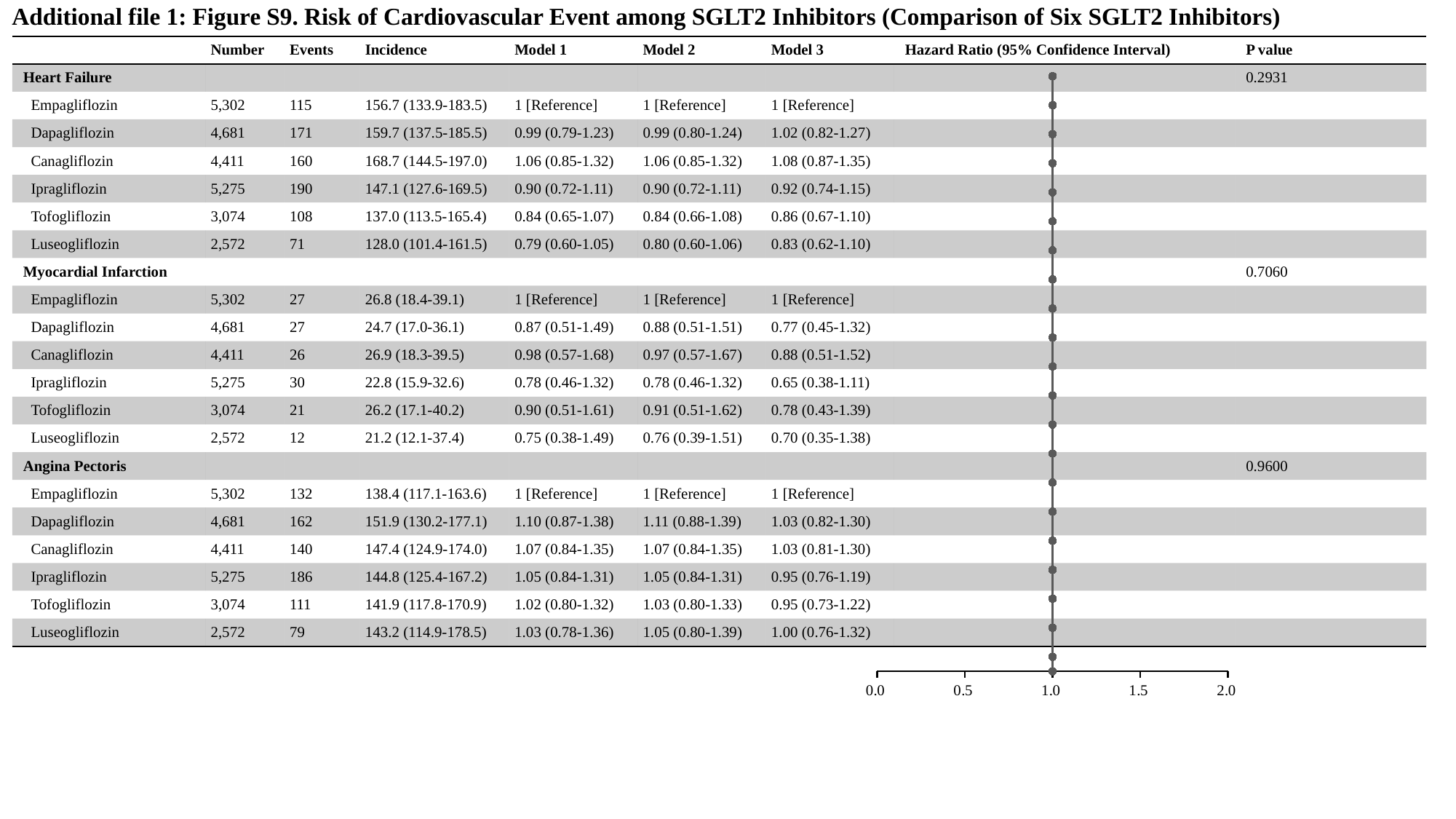

Additional file 1: Figure S9. Risk of Cardiovascular Event among SGLT2 Inhibitors (Comparison of Six SGLT2 Inhibitors)
| | Number | Events | Incidence | Model 1 | Model 2 | Model 3 | Hazard Ratio (95% Confidence Interval) | P value |
| --- | --- | --- | --- | --- | --- | --- | --- | --- |
| Heart Failure | | | | | | | | 0.2931 |
| Empagliflozin | 5,302 | 115 | 156.7 (133.9-183.5) | 1 [Reference] | 1 [Reference] | 1 [Reference] | | |
| Dapagliflozin | 4,681 | 171 | 159.7 (137.5-185.5) | 0.99 (0.79-1.23) | 0.99 (0.80-1.24) | 1.02 (0.82-1.27) | | |
| Canagliflozin | 4,411 | 160 | 168.7 (144.5-197.0) | 1.06 (0.85-1.32) | 1.06 (0.85-1.32) | 1.08 (0.87-1.35) | | |
| Ipragliflozin | 5,275 | 190 | 147.1 (127.6-169.5) | 0.90 (0.72-1.11) | 0.90 (0.72-1.11) | 0.92 (0.74-1.15) | | |
| Tofogliflozin | 3,074 | 108 | 137.0 (113.5-165.4) | 0.84 (0.65-1.07) | 0.84 (0.66-1.08) | 0.86 (0.67-1.10) | | |
| Luseogliflozin | 2,572 | 71 | 128.0 (101.4-161.5) | 0.79 (0.60-1.05) | 0.80 (0.60-1.06) | 0.83 (0.62-1.10) | | |
| Myocardial Infarction | | | | | | | | 0.7060 |
| Empagliflozin | 5,302 | 27 | 26.8 (18.4-39.1) | 1 [Reference] | 1 [Reference] | 1 [Reference] | | |
| Dapagliflozin | 4,681 | 27 | 24.7 (17.0-36.1) | 0.87 (0.51-1.49) | 0.88 (0.51-1.51) | 0.77 (0.45-1.32) | | |
| Canagliflozin | 4,411 | 26 | 26.9 (18.3-39.5) | 0.98 (0.57-1.68) | 0.97 (0.57-1.67) | 0.88 (0.51-1.52) | | |
| Ipragliflozin | 5,275 | 30 | 22.8 (15.9-32.6) | 0.78 (0.46-1.32) | 0.78 (0.46-1.32) | 0.65 (0.38-1.11) | | |
| Tofogliflozin | 3,074 | 21 | 26.2 (17.1-40.2) | 0.90 (0.51-1.61) | 0.91 (0.51-1.62) | 0.78 (0.43-1.39) | | |
| Luseogliflozin | 2,572 | 12 | 21.2 (12.1-37.4) | 0.75 (0.38-1.49) | 0.76 (0.39-1.51) | 0.70 (0.35-1.38) | | |
| Angina Pectoris | | | | | | | | 0.9600 |
| Empagliflozin | 5,302 | 132 | 138.4 (117.1-163.6) | 1 [Reference] | 1 [Reference] | 1 [Reference] | | |
| Dapagliflozin | 4,681 | 162 | 151.9 (130.2-177.1) | 1.10 (0.87-1.38) | 1.11 (0.88-1.39) | 1.03 (0.82-1.30) | | |
| Canagliflozin | 4,411 | 140 | 147.4 (124.9-174.0) | 1.07 (0.84-1.35) | 1.07 (0.84-1.35) | 1.03 (0.81-1.30) | | |
| Ipragliflozin | 5,275 | 186 | 144.8 (125.4-167.2) | 1.05 (0.84-1.31) | 1.05 (0.84-1.31) | 0.95 (0.76-1.19) | | |
| Tofogliflozin | 3,074 | 111 | 141.9 (117.8-170.9) | 1.02 (0.80-1.32) | 1.03 (0.80-1.33) | 0.95 (0.73-1.22) | | |
| Luseogliflozin | 2,572 | 79 | 143.2 (114.9-178.5) | 1.03 (0.78-1.36) | 1.05 (0.80-1.39) | 1.00 (0.76-1.32) | | |
### Chart
| Category | | | | |
|---|---|---|---|---|

## Slide 10
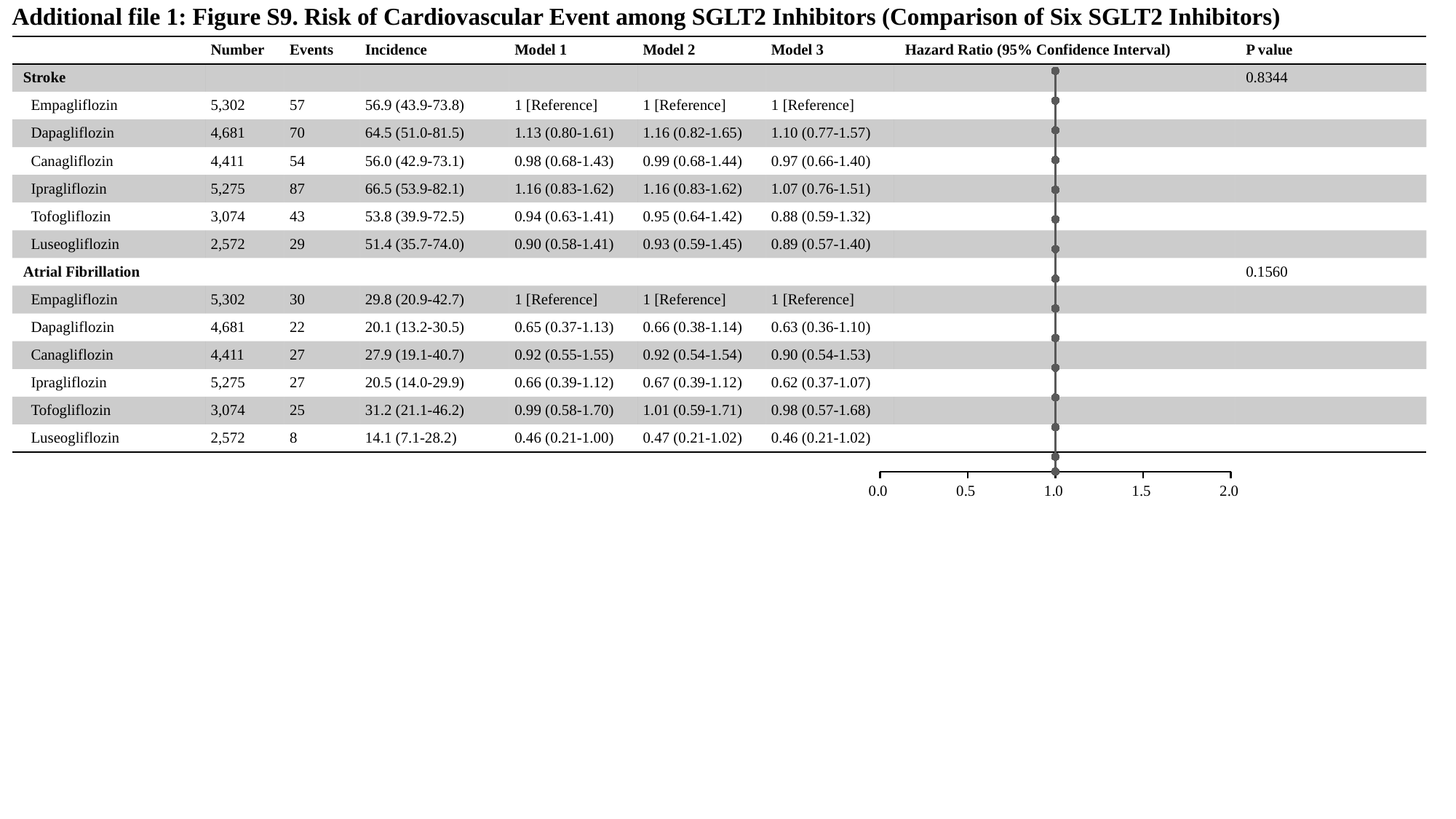

Additional file 1: Figure S9. Risk of Cardiovascular Event among SGLT2 Inhibitors (Comparison of Six SGLT2 Inhibitors)
| | Number | Events | Incidence | Model 1 | Model 2 | Model 3 | Hazard Ratio (95% Confidence Interval) | P value |
| --- | --- | --- | --- | --- | --- | --- | --- | --- |
| Stroke | | | | | | | | 0.8344 |
| Empagliflozin | 5,302 | 57 | 56.9 (43.9-73.8) | 1 [Reference] | 1 [Reference] | 1 [Reference] | | |
| Dapagliflozin | 4,681 | 70 | 64.5 (51.0-81.5) | 1.13 (0.80-1.61) | 1.16 (0.82-1.65) | 1.10 (0.77-1.57) | | |
| Canagliflozin | 4,411 | 54 | 56.0 (42.9-73.1) | 0.98 (0.68-1.43) | 0.99 (0.68-1.44) | 0.97 (0.66-1.40) | | |
| Ipragliflozin | 5,275 | 87 | 66.5 (53.9-82.1) | 1.16 (0.83-1.62) | 1.16 (0.83-1.62) | 1.07 (0.76-1.51) | | |
| Tofogliflozin | 3,074 | 43 | 53.8 (39.9-72.5) | 0.94 (0.63-1.41) | 0.95 (0.64-1.42) | 0.88 (0.59-1.32) | | |
| Luseogliflozin | 2,572 | 29 | 51.4 (35.7-74.0) | 0.90 (0.58-1.41) | 0.93 (0.59-1.45) | 0.89 (0.57-1.40) | | |
| Atrial Fibrillation | | | | | | | | 0.1560 |
| Empagliflozin | 5,302 | 30 | 29.8 (20.9-42.7) | 1 [Reference] | 1 [Reference] | 1 [Reference] | | |
| Dapagliflozin | 4,681 | 22 | 20.1 (13.2-30.5) | 0.65 (0.37-1.13) | 0.66 (0.38-1.14) | 0.63 (0.36-1.10) | | |
| Canagliflozin | 4,411 | 27 | 27.9 (19.1-40.7) | 0.92 (0.55-1.55) | 0.92 (0.54-1.54) | 0.90 (0.54-1.53) | | |
| Ipragliflozin | 5,275 | 27 | 20.5 (14.0-29.9) | 0.66 (0.39-1.12) | 0.67 (0.39-1.12) | 0.62 (0.37-1.07) | | |
| Tofogliflozin | 3,074 | 25 | 31.2 (21.1-46.2) | 0.99 (0.58-1.70) | 1.01 (0.59-1.71) | 0.98 (0.57-1.68) | | |
| Luseogliflozin | 2,572 | 8 | 14.1 (7.1-28.2) | 0.46 (0.21-1.00) | 0.47 (0.21-1.02) | 0.46 (0.21-1.02) | | |
### Chart
| Category | | | | |
|---|---|---|---|---|
